# Sedimentological and micropaleontological characteristics of tsunami deposits associated with the 2024 Noto Peninsula earthquake

**DOI:** 10.1038/s41598-025-90945-w

**Published:** 2025-03-21

**Authors:** Yumi Shimada, Dan Matsumoto, Koichiro Tanigawa, Yuki Sawai

**Affiliations:** Geological Survey of Japan, National Institute of Advanced Industrial Science and Technology (AIST), AIST Tsukuba Central 7, 1-1-1 Higashi, Tsukuba, Ibaraki 305-8567 Japan

**Keywords:** The 2024 Noto Peninsula earthquake, Tsunami deposits, Diatom, Sedimentary structures, Vented sediment, Natural hazards, Palaeontology, Sedimentology

## Abstract

This study reports sedimentological and paleontological features of deposits left by the 2024 Noto Peninsula tsunami in Suzu City, Japan. Tsunami deposits were found up to 70 m inland from the post-tsunami shoreline along our transect. The tsunami deposits were collected at five locations for observation by Soft X-ray and CT images, grain-size analysis, and diatom analysis. Soft X-ray and CT images identified that the five stratigraphic units (Units 1–5) at the most seaward location (SZ1) and deposits with faint laminae at the other locations (SZ2–4). Grain-size analysis showed that the tsunami deposits generally composed of fine to very fine sand at all sampled locations. At SZ1, Unit 3 exhibits climbing ripples with their leeside seaward. The ripple tops were probably dragged seaward. The eroded upper contact of Unit 4 implies yet another current at SZ1. Diatom assemblages within the tsunami deposits are dominated by marine and brackish species, except Unit 4 at SZ1 with more than 30% freshwater terrestrial species. Diatom assemblages in the tsunami deposits, vented sediments, and beach sand suggest that the SZ1 tsunami deposit was derived from both terrestrial and marine sources, while the main source was the coastal beach at the other locations.

## Introduction

Sediments left by tsunamis, known as tsunami deposits, have been used to reconstruct the history of infrequent catastrophic tsunamis worldwide^1–7^. Tsunami deposits aid in reconstructing inundation areas^8,9^, recurrence intervals^10–13^, rupture areas as tsunami sources^14,15^, and flow conditions^16–19^. Tsunami deposits are first identified as coarse grain layers within fine grain sediments formed in calm environments, and then diagnosed by empirical and theoretical criteria such as thickness changes, sedimentary structures, and paleontological features^20–22^ to distinguish them from deposits formed by other types of events (e.g., floods and storms). In the last few decades prior to the 2004 Sumatra earthquake, tsunami deposits have been regarded to have relatively simple sedimentary features (e.g., massive or graded, lower sharp contact, and mud cap on the top)^23–25^ and uniform characteristics of micropaleontology, especially in diatom assemblages (e.g., fragmented valves and marine or mixed diatom assemblages)^1,24,26^. However, observation of tsunami deposits associated with the 2004 Sumatra earthquake overturned such recognitions and provided reports on various sedimentary features and diverse diatom assemblages within the tsunami deposits^27,28^. More recently, the 2011 Tohoku tsunami gave us additional insights of features of tsunami deposits^29–31^. To clarify the causes of such variations and sedimentary processes of tsunami deposits, comprehensive research will be needed to describe modern tsunami deposits covering various geological and geomorphological settings and probably also various magnitudes of tsunami inundation.

**Fig. 1:**
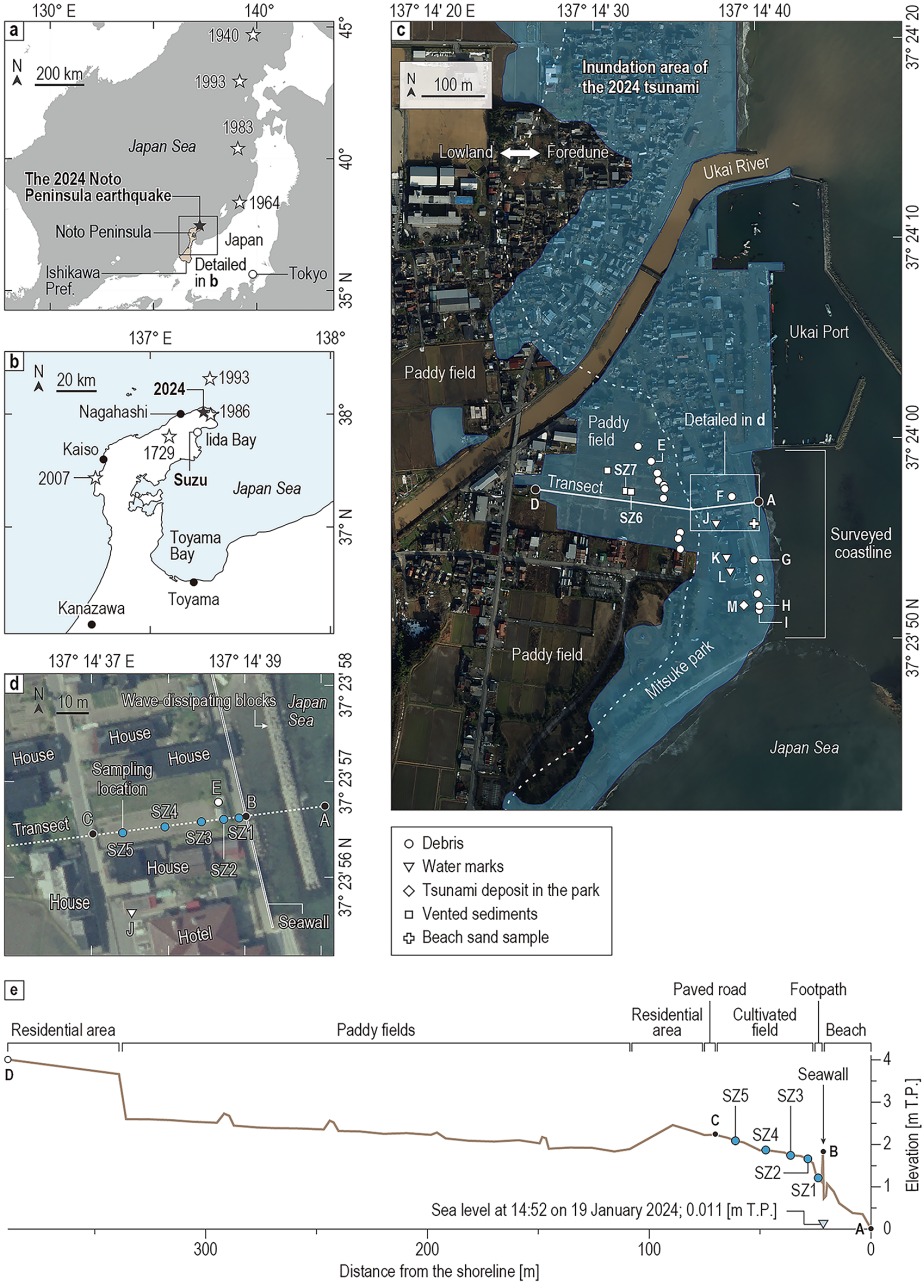
(**a**) Location of the Noto Peninsula, Japan. The black star represents the epicenter of the 2024 Noto Peninsula earthquake, and the white stars represent the epicenters of past earthquakes estimated to have been Mw ≥ 7.0. Generic Mapping Tools (GMT)^69^, a free software package, was partially used. (**b**) Map of the Noto Peninsula showing the location of Suzu City and the epicenters of historical earthquakes. The white stars represent the epicenters of recent and historical earthquakes around Noto Peninsula. (**c**) Aerial photograph of the southeastern part of Suzu City taken the day after the earthquake (2 January 2024) with the locations of debris, water marks, a tsunami deposit at a separate location from the study site, and vented sediments found in our field survey. The debris locations indicate the inland limit of the distribution of artificial debris greater than ~ 30 cm, and there was smaller debris scattered on the seaward side. Detailed information on each location is provided in Table 1. The photograph was taken and provided by the Geospatial Information Authority of Japan (GSI). Inundation areas were estimated by the GSI^41^. (**d**) Sediment sampling locations and setting of our study site. The photograph was taken by the GSI on 15 May 2010 (photograph number: CCB20101X-C09-25). (**e**) Topographic profile and land use along transect A–D in (**c**). Elevations are expressed with respect to mean sea level at Tokyo Bay (Tokyo Peil, T.P.).

In this study, we described and collected tsunami deposits associated with the 2024 earthquake at Suzu City on the northeast coast of the Noto Peninsula, Japan (Fig. 1), with the aim of documenting the sedimentary and micropaleontological features of the deposits. This study of a modern tsunami deposit will contribute to improved interpretation of paleo-tsunami deposits in this region.

## The 2024 Noto Peninsula earthquake

On 1 January 2024, the Noto Peninsula earthquake (Mj 7.6 on the Japan Meteorological Agency magnitude scale) occurred along the northern part of the Noto Peninsula, Ishikawa Prefecture, Japan^32^ (Fig. 1). This earthquake was caused by the rupture of a reverse fault with an NE–SW axis. The seismic activity area of the aftershocks extended for approximately 150 km in the NE–SW direction^32^. The earthquake caused strong ground motion and severe damage at the coast and in the surrounding areas of the peninsula. GNSS observations detected crustal deformation along the northwestern coast of the peninsula^33^ including a 3.6 m uplift in Kaiso (Fig. 1b)^34^, although there was little vertical deformation around the study site^33^. The associated tsunami source was estimated to be a fault approximately 100 km long as assessed from joint inversion modeling using GNSS and tide and wave gauge data^35^.

## Study area

### Geological and geomorphological setting

The study site is located south of the mouth of the Ukai River, which flows into Iida Bay (Fig. 1b–d). A Holocene foredune stretches north and south. The lowlands behind the foredune are filled with Holocene alluvial deposits^36^. Paddy fields and other small cultivated fields, houses, and narrow footpaths are isolated from the sea by an artificial seawall of 1.8 m T.P. (Tokyo Peil; the mean sea level in Tokyo Bay, the reference level of elevation in Japan, the annual mean sea level in 2023 was approximately + 24 cm T.P. at the nearest tide station at Nagahashi^37^) (Fig. 1b,e). The bathymetry offshore of the study area is 7 m at ~ 500 m and 11 m at ~ 1000 m from the shore^38^.

### Tsunami effects in the study area

There are reports about inundation by the tsunami associated with the 2024 Noto Peninsula earthquake in the study area. An eyewitness account stated that at least two tsunami waves reached the residential area on the south side of the Ukai River^39^. Japan Meteorological Agency announced that a post-tsunami survey of watermarks reported the tsunami trace height reached 2.5–3.0 m T.P. at Ukai Port and Mitsuke Park^40^. The Geospatial Information Authority of Japan estimated the inundation distance along our transect, which was interpreted from satellite images after the earthquake, to be approximately 350 m from the post-tsunami shoreline^41^. UAV images taken about 2 weeks after the earthquake record tsunami deposits spreading up to about 100 m from the shoreline^42^. Eyewitnesses reported that tsunami waves ran up and left debris at least 500 m along the Hannya River, which is located about 500 m north of the Ukai River^42^.

The tsunami waveforms were not recorded because the nearest tide gauge at Nagahashi (Fig. 1b), on the northwestern part of the Noto Peninsula, did not work immediately after the earthquake as a result of coastal uplift associated with the event^43^. A tsunami propagation and inundation model estimated the time history of offshore wave level and onshore inundation depth in the coastal area around the Ukai River^44^. The second wave, approximately 30 min after the earthquake, was estimated to have caused the highest water level, and the inundation depth of the following tsunami reached approximately 1.5 m. The modeled inundation distance of the tsunami extended approximately 400 m inland from the shoreline along our transect^44^.

**Fig. 2:**
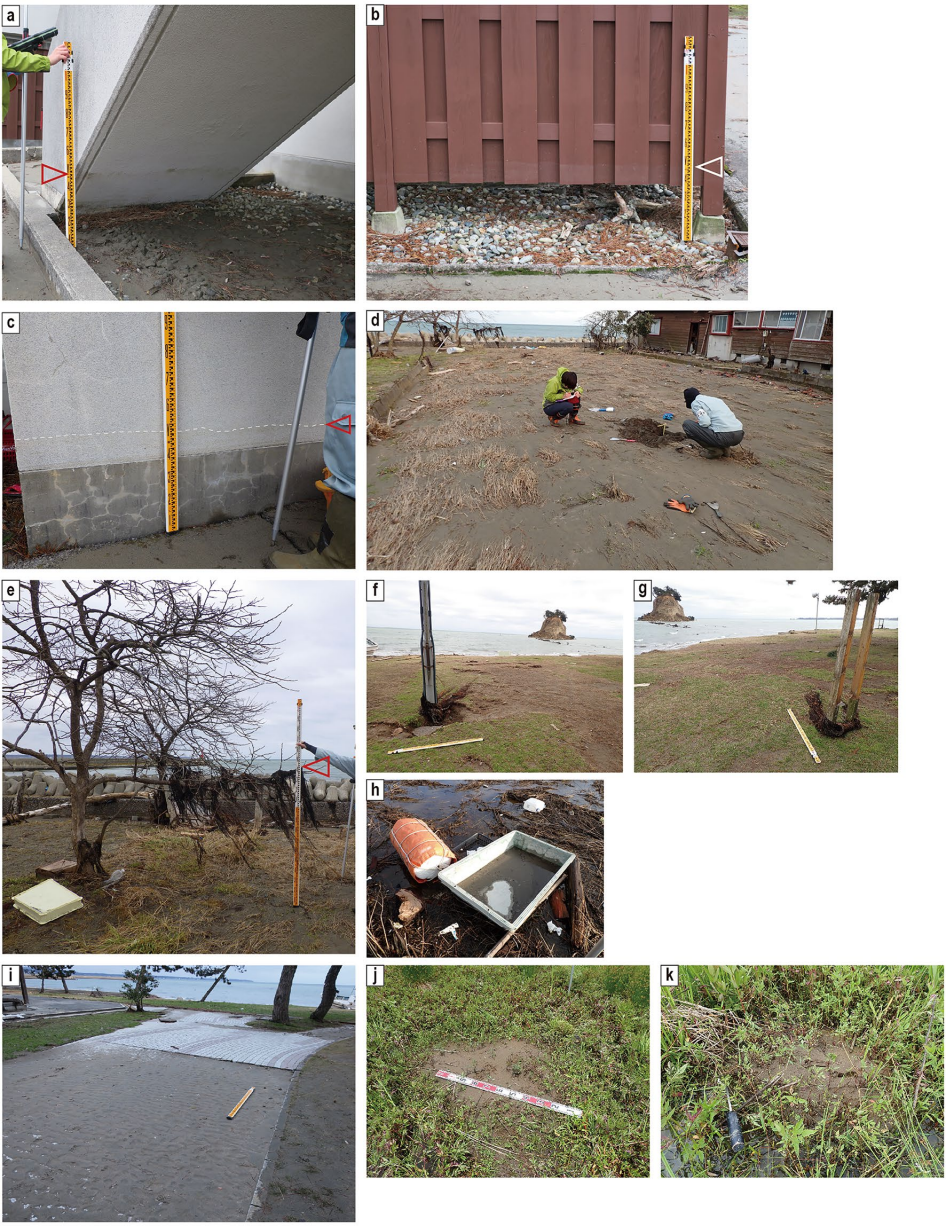
Photographs of the field survey locations around the study area in Suzu City. All the locations are indicated in Fig. 1c. (**a**) Watermark at location L. (**b**) Watermark at location K. (**c**) Watermark at location J. (**d**) Our study site. (**e**) Debris at our study site (location F). Grass trapped 140 cm from the ground. (**f**) Grass trapped by a pole at location H. (**g**) Grass trapped by a pole at location I. (**h**) Debris at location E. (**i**) Tsunami deposit with ripples at location M. (**j**) Vented sediments at location SZ6. (**k**) Vented sediments at location SZ7.

In this study, we examined traces of the 2024 tsunami such as debris, watermarks, tsunami deposits, and liquefaction-induced vented sediments. We also carried out a trench survey, described the tsunami deposits in a small cultivated field and on a footpath (Figs. 1e and 2d), and collected box samples at five locations for sedimentary and diatom analyses.

## Results and discussion

**Fig. 3:**
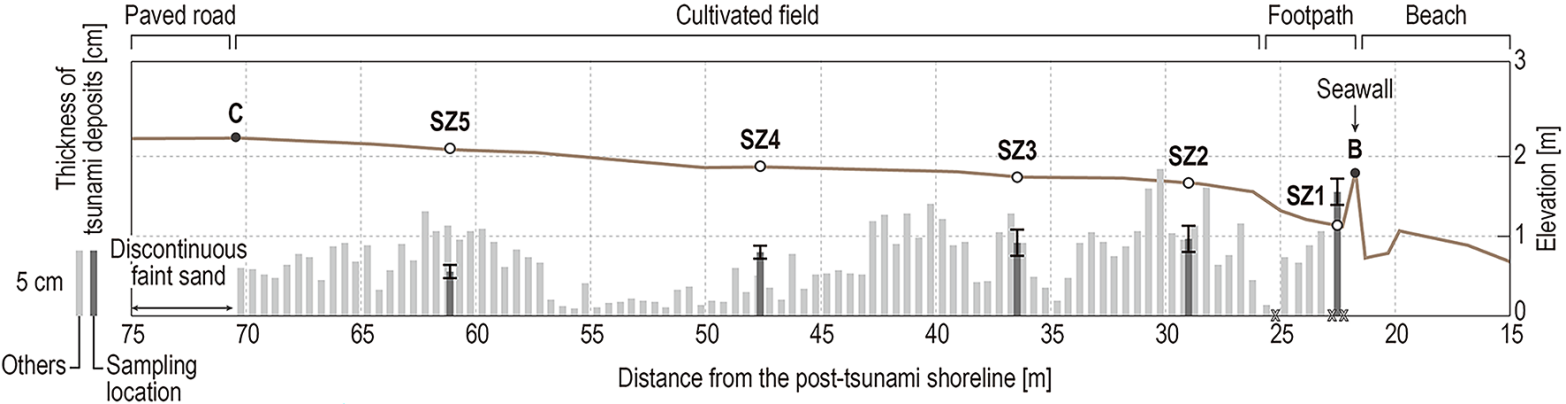
Thickness of tsunami deposits and distance from the post-tsunami shoreline along transect A–D on Fig. 1d. The brown line represents the elevation of the ground surface along the transect (measured 19th January 2024). Black bars show tsunami deposit thicknesses at sites from which the analyzed samples were taken. The error bars represent ranges of thicknesses within sampling pits. Light gray bars represent thickness of the tsunami deposits at locations where only deposit thickness was measured. Cross marks represent locations where tsunami deposits were absent because of the presence of artificial structures (e.g., blocks, road curbs).

**Fig. 4:**
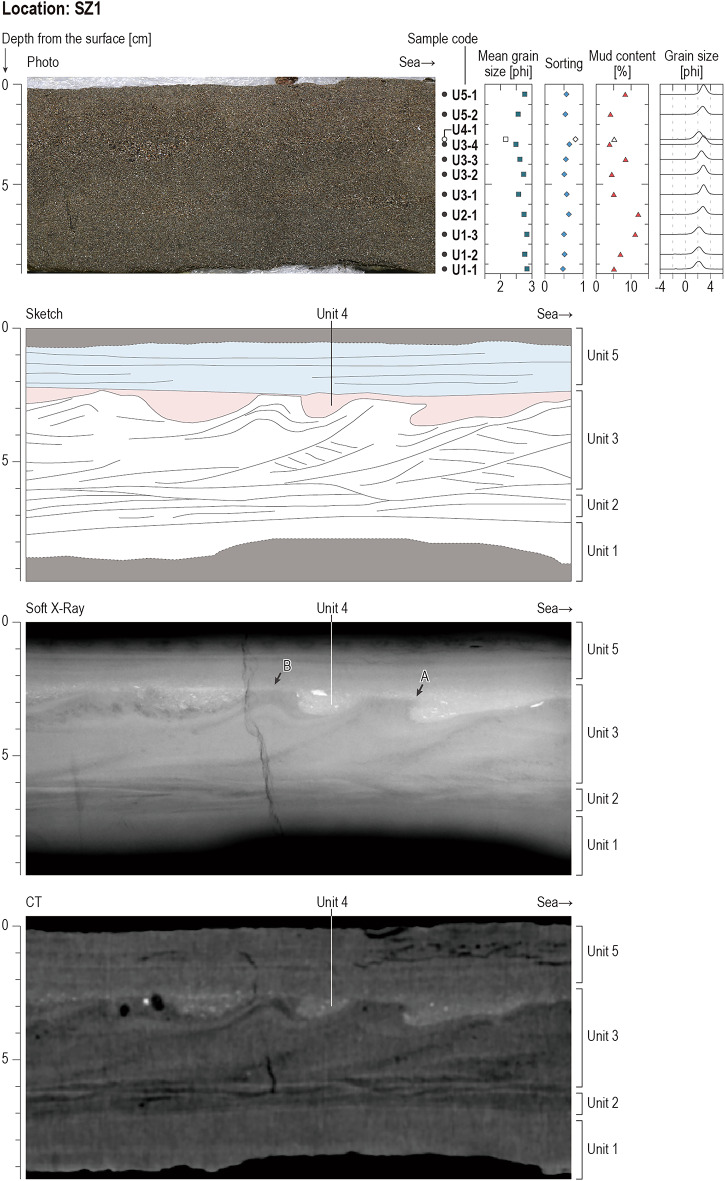
A photograph, a CT image, a soft X-ray image, a sketch, and graphs showing vertical changes in mean grain size, sorting, mud content, grain-size distribution at location SZ1. The mean grain size, sorting, mud content, and grain-size distribution of the sample from Unit 4 (U4-1) are represented by black symbols, to distinguish this sample from others from a similar depth. The specific sampling horizons from U1-1 to U5-2 are provided in the Supplementary Data S5.

**Fig. 5:**
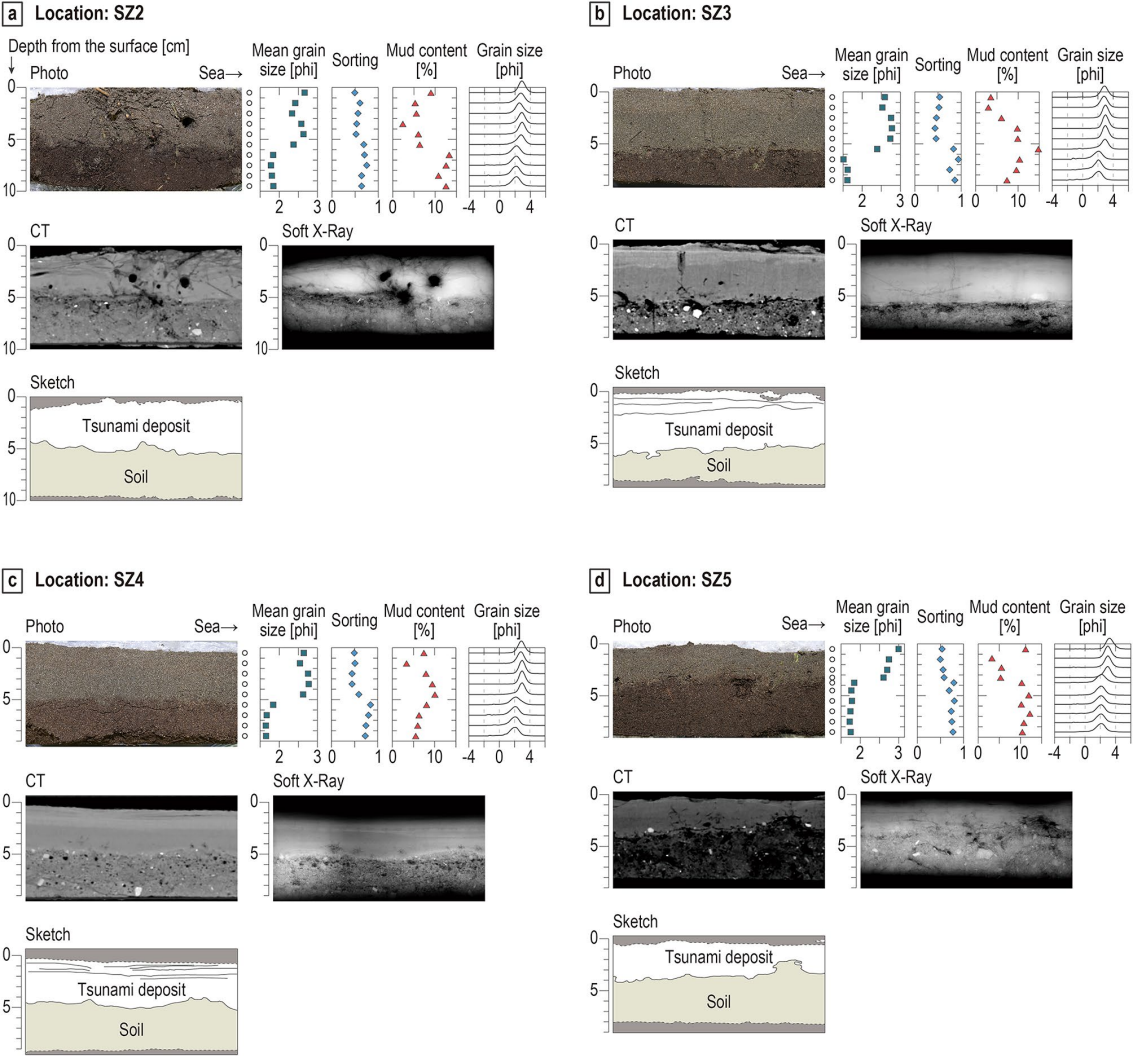
Photographs, CT images, soft X-ray images, sketches, and graphs showing vertical changes in mean grain size, sorting, mud content, and grain-size distribution at localities (**a**) SZ2, (**b**) SZ3, (**c**) SZ4, and (**d**) SZ5.

**Fig. 6:**
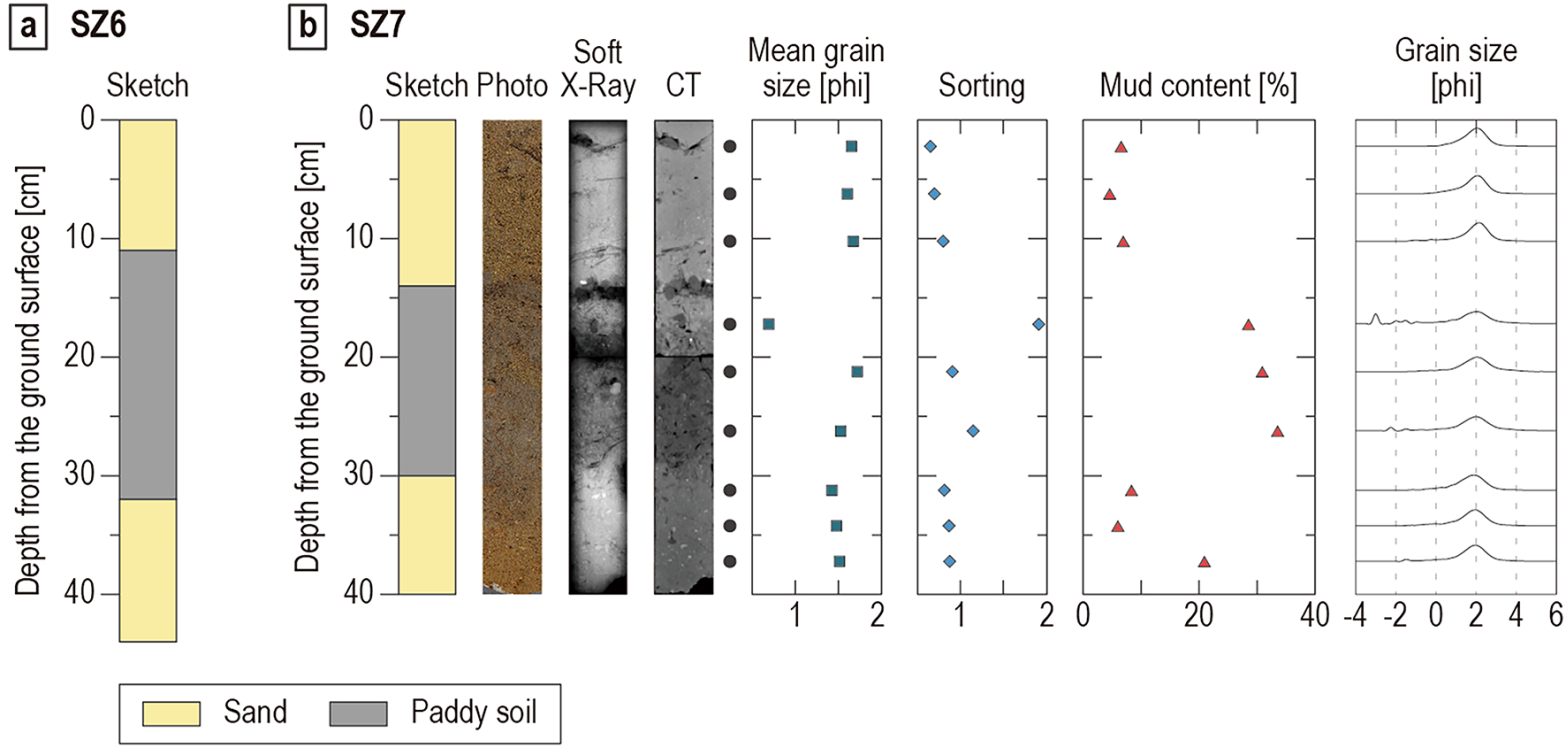
(**a**) Stratigraphic column at location SZ6. (**b**) Stratigraphic column, photograph, soft X-ray image, CT image, and results of grain-size analysis at location SZ7. The discontinuity exists at a depth of 20 cm below the ground surface, as the core sediment was divided into two acrylic cases at depths of 0–20 cm and 20–40 cm for CT and soft X-ray imaging.

Seventeen days after the earthquake and tsunami, we carried out a field survey and recognized extensive evidence of tsunami inundation, such as the presence of debris and watermarks around the Ukai River mouth (Fig. 1c). We also identified tsunami deposits and liquefaction-induced vented sediments, and surveyed the distribution of tsunami deposits along approximately 300 m of coastline (the surveyed coastline in Figs. 1c, 3, 4, 5 and 6). Tsunami deposits as layered sediments were observed only on a foot path along the seawall, in a cultivated field (Fig. 2d), and in the Mitsuke park (Fig. 2i). At five representative locations (SZ1–SZ5), we dug small pits and trenches, described the tsunami deposits, and collected box samples for detailed observation and analyses in the laboratory. We also measured the changes in thickness of the tsunami deposits at random locations along transect A–D (Fig. 1c).

### Observed inundation height, debris, and liquefaction features around the study site

The height of watermarks allowed us to estimate the tsunami flow depth. Fresh watermarks around transect A–D (Fig. 1c) were 45–47 cm above ground level (Fig. 2a–c and Table 1).

**Table 1:** List of debris, water marks, a tsunami deposit, and vented sediments around the study site. Latitudes and longitudes marked with asterisks were obtained based on GSM maps released by the Geospatial Information Authority of Japan^70^.

Location		Object	Details
Latitude	Longitude		
37° 23′ 59.78″ N	137° 14′ 32.45″ E	Debris	Basket and bucket
37° 23′ 58.99″ N	137° 14′ 33.29″ E	Debris	Tub and float (E in Figs. 1c, 2h)
37° 23′ 58.37″ N	137° 14′ 33.68″ E	Debris	Bucket
37° 23′ 57.97″ N	137° 14′ 33.74″ E	Debris	Float
37° 23′ 57.78″ N	137° 14′ 34.09″ E	Debris	Float
37° 23′ 57.61″ N	137° 14′ 34.10″ E	Debris	Refrigerator
37° 23′ 56.95″ N	137° 14′ 33.98″ E	Debris	Polystyrene box
37° 23′ 55.33″ N	137° 14′ 35.09″ E	Debris	Float
37° 23′ 55.01″ N	137° 14′ 35.02″ E	Debris	Part of a wooden fence
37° 23′ 54.49″ N	137° 14′ 35.16″ E	Debris	Flowerpot
37° 23′ 56.72″ N	137° 14′ 38.59″ E	Debris	Grass on a tree (140 cm from the ground, F in Figs. 1c, 2e)
37° 23′ 52.99″ N*	137° 14′ 38.47″ E*	Water mark	45 cm from the ground (L in Figs. 1c, 2a)
37° 23′ 53.75″ N	137° 14′ 38.16″ E	Water mark	45 cm from the ground (K in Figs. 1c, 2b)
37° 23′ 55.65″ N	137° 14′ 37.42″ E	Water mark	47 cm from the ground (J in Figs. 1c, 2c)
37° 23′ 53.89″ N*	137° 14′ 39.71″ E*	Debris	Grass on a tree (147 cm from the ground, G in Fig. 1c)
37° 23′ 53.67″ N*	137° 14′ 39.65″ E*	Debris	Table
37° 23′ 51.58″ N	137° 14′ 39.2″ E	Tsunami deposit	Ripples observed (M in Figs. 1c, 2i)
37° 23′ 52.05″ N*	137° 14′ 39.96″ E*	Debris	Grass trapped by a tree
37° 23′ 51.42″ N*	137° 14′ 40.17″ E*	Debris	Grass trapped by a pole (H in Figs. 1c, 2f)
37° 23′ 51.07″ N*	137° 14′ 40.32″ E*	Debris	Grass trapped by a pole (I in Figs. 1c, 2g)
37° 23′ 58.30″ N*	137° 14′ 30.30″ E*	Vented sediments	~ 50 cm in diameter
37° 23′ 57.28″ N	137° 14′ 31.83″ E	Vented sediments	~ 135 cm in diameter (SZ6 in Figs. 1c; 2j)
37° 23′ 57.34″ N	137° 14′ 31.41″ E	Vented sediments	~ 55 cm in diameter (SZ7 in Figs. 1c; 2k)

Debris such as human-produced objects, garbage, and drifting plants were observed, and the inland limit of the artificial debris greater than ~ 30 cm in size was recognized about 160 m inland from the shoreline (Figs. 1c, 2h and Table 1). Plant debris trapped on trees at locations F and G (Fig. 1c) showed inundation heights of 140 cm and 147 cm above ground level, respectively (Fig. 2e and Table 1). Plant material trapped by trees and poles near the coast and leaning plants in the paddy fields had been dragged in the seaward direction, thus indicating the last flow direction of the tsunami wave (Supplementary Data S1).

We observed liquefaction features at many locations in the paddy fields (Fig. 2j,k, and Table 1) near our transect. The diameters of vented liquefied sand ranged from 30 to 135 cm. We collected samples from the vented sediments as reference samples for comparison with the tsunami deposits. Detailed descriptions of the vented sediments at locations SZ6 and SZ7 are provided below (see section “Sedimentology and diatom assemblages of vented sediments”).

### Distribution and thickness of the 2024 tsunami deposits

The thickness of the tsunami deposits measured every half meter along transect A–D (22–70 m inland from the post-tsunami shoreline) ranged from 0.6 to 11.2 cm (Fig. 3), with the maximum value recorded at 30 m and the minimum at 56 m inland from the shoreline. The thickness of the tsunami deposits varies along the whole transect. Discontinuous faint sand sediments were observed up to 75 m from the shoreline on the paved road inland of the sampled cultivated field. Tsunami deposits were also recognized in the Mitsuke park about 150 m south of the study site (Fig. 2i and Table 1), but we did not measure changes in the thickness of this deposit because the sediments sparsely cover the ground surface and do not form a clear layer.

We observed a substantial difference between the tsunami inundation distance and the extent of tsunami deposits along our studied transect. The tsunami inundation limit inferred by us from the distribution of debris that we identified in the field and the limit estimated by using aerial photographs and satellite images by the Geospatial Information Authority of Japan^41^ were about 160 m and 350 m inland from the shoreline, respectively. In contrast, the tsunami deposit is limited to a maximum of only 70 m from the shoreline along the transect, corresponding to 44% and 20% of the inundation distance estimated by this study and by the Geospatial Information Authority of Japan, respectively. Such a difference in distance between marine water inundation and tsunami traces (e.g., debris and sediments) has been previously reported after recent great tsunamis, for example, the 2004 Sumatra and 2011 Tohoku events^31,45–49^. Tsunami sedimentation is affected by many different factors: topographic effects of small-scale irregularities (e.g., mounts and depressions); relatively large-scale changes in topography (e.g., changes in slope), decreasing water flow velocities as the wave moves far inland; and seawalls or other obstacles that cause quick changes in the velocity and direction of the current. With regard to this low ratio of the tsunami deposit distribution to inundation distance, a relatively small tsunami such as the 2024 Noto event might be affected more by small-scale topography and obstacles than by large-scale topography, whereas great tsunamis are probably more affected by large-scale topography (e.g., topographic gradients over hundreds or thousands of meters). We note that an additional reason for differences between inundation and tsunami sediments might be an irregular source of sediment (e.g., vented sediments).

### Sedimentology and diatom assemblages of tsunami deposits

**Fig. 7:**
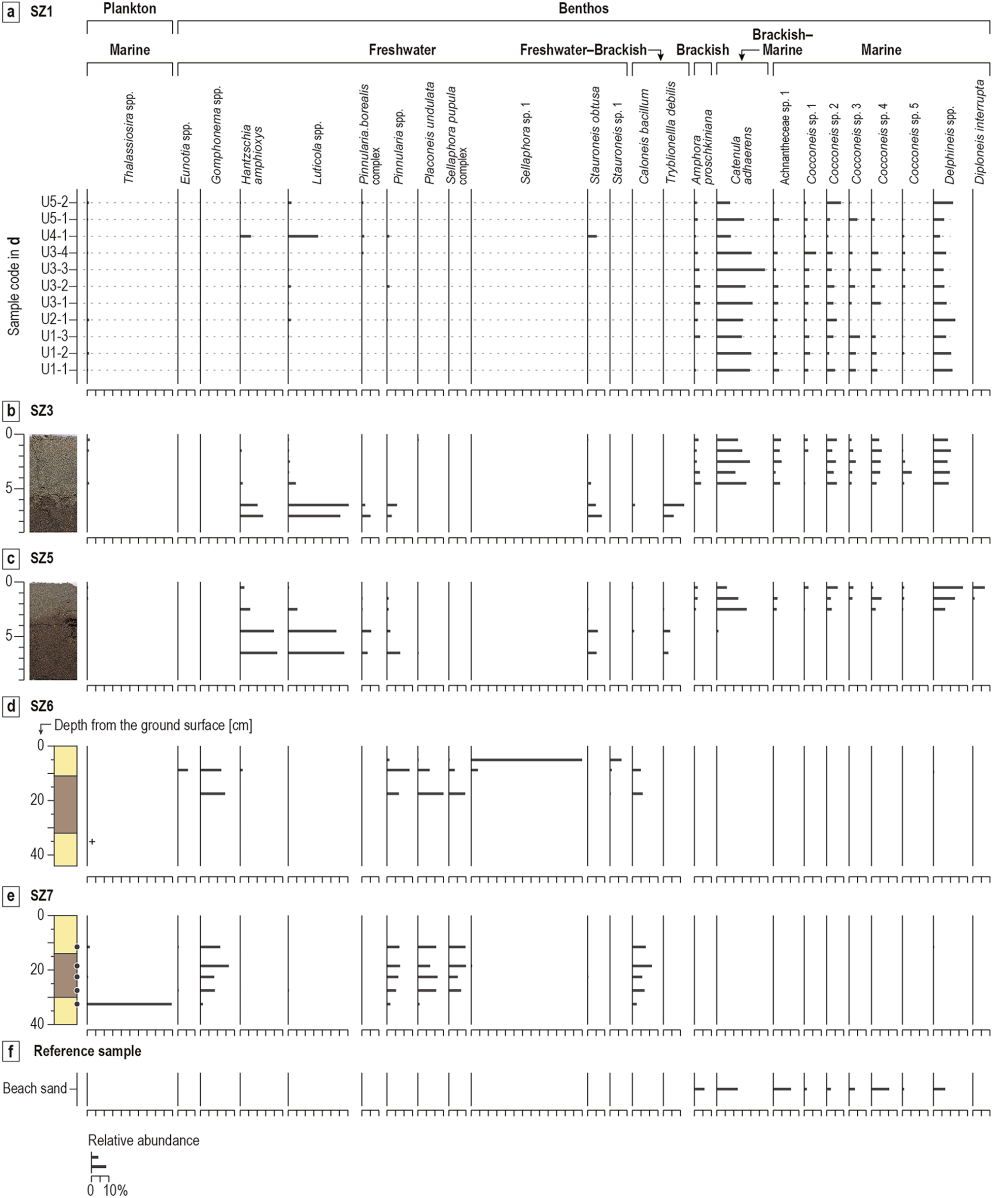
(**a**) Fossil diatom analysis results for location SZ1. The diagram shows selected taxa with a relative abundance of 5% in at least one sample. The scale of relative abundance is the same in (**a**–**f**). The specific sampling horizons from U1-1 to U5-2 are provided in the Supplementary Data S5. (**b**) Fossil diatom analysis results for location SZ3. (**c**) Fossil diatom analysis results for location SZ5. (**d**) Fossil diatom analysis results for vented sediments and paddy field soil at SZ6. (**e**) Fossil diatom analysis results for vented sediments, paddy field soil, and the light brown sand underlying paddy soil at SZ7. (**f**) Diatom assemblages within beach sand.

Tsunami deposits were recognized on a paved footpath at SZ1 and above a cultivated field at the other four sampling locations SZ2–SZ5 (Fig. 1d,e). The sand fraction of the tsunami deposits consists mainly of very fine to fine sand (Figs. 4, 5, & Supplementary Data S2). The diatom assemblages within the tsunami sand at SZ1, SZ3, and SZ5 are composed of brackish, brackish–marine, and marine species, except in one unit at SZ1. The sedimentary and micropaleontological characteristics and inferred depositional processes of the tsunami deposits at each location are described below and illustrated in Figs. 4, 5, and 7.

#### Location SZ1

Location SZ1, the most seaward sampling point of this study, is situated on a footpath. At this location, the tsunami deposit is 9–10 cm thick and consists mainly of five units (Units 1–5 from bottom to top, Fig. 4 and Supplementary Data S3). During the field survey, we defined only two units—the uppermost part of the succession (Unit 5) and the lower part of the succession (all other units)—on the basis of the sediment color (dark gray in Units 1–4 and brown in Unit 5). Subsequently, in the lab, we distinguished five units based on their sedimentary structures in computed tomography (CT) and soft X-ray images (Fig. 4, Supplementary Datas S3 and S4). The lowest unit, **Unit 1,** is a 3-cm-thick (approximately 7–10 cm from the top of the deposit) fine sand exhibiting faint parallel laminations. Mean grain size of the sand fraction in Unit 1 is 2.77–2.84 phi. **Unit 2** is a 1-cm-thick (approximately 6–7 cm from the surface) fine sand distinguished by the highest mud content at this location (12.06 wt. %) and the presence of parallel and wavy laminae. Mean grain size of the sand fraction in Unit 2 is 2.75 phi. This unit thins seaward on CT and soft-X ray images. **Unit 3** is a 4-cm-thick (approximately 2–6 cm from the surface) fine sand, exhibiting prominent climbing ripples with their leesides to seaward. The top crests of the climbing ripples in this unit are flattened and, possibly truncated in the seaward direction. A wisp of Unit 3 sticks into Unit 4 (Arrow A in Fig. 4). Grain-size analysis of Unit 3 showed a slight change in the mean grain size of the sand fraction within fine sand (between 2.49 and 2.74 phi in mean grain size). Units 1–3, as a whole, exhibit a single inverse grading trend (Fig. 4 and Supplementary Data S2). **Unit 4** is of variable thickness, filling the ripple-trough depressions at the top of Unit 3, including the flame-structure geometry. Unit 4 is a poorly sorted fine sand containing many unidentified shell fragments and sponge spicules. The mean grain size of the sand fraction in Unit 4 (2.16 phi) is coarser than those of other units (2.49–2.84 phi, average 2.69 phi). Soft X-ray and CT images show that Unit 4 is partly discontinuous; the most seaward crest of 3 is covered by Unit 4, which is further overlain by Unit 5, but another Unit 3 crest is directly covered by Unit 5 (Arrow B in Fig. 4). **Unit 5**, the uppermost unit, is a 2-cm thick (approximately 0–2 cm from the surface) fine sand exhibiting parallel laminations. Mean grain size of the sand fraction in Unit 5 is 2.56–2.77 phi. This unit shows upward-fining and a distinct lower contact.

The diatom assemblages in 11 samples (U1-1 to U5-2 from bottom to top in Supplementary data S5) from the tsunami deposits at location SZ1 are dominated mostly by brackish–marine and marine taxa, except for sample U4-1 (Fig. 7a and Supplementary Data S6). A brackish–marine species, *Catenula adhaerens*, is the dominant species in the nine samples from Units 1–3, and 5 (samples U1-1 to U3-4 and sample U5-1). *C. adhaerens* is still abundant in the sample from the top of the deposit (U5-2), but the first dominant taxon in this sample is marine *Delphineis* spp. Sample U4-1 is characterized by a mixed assemblage of freshwater terrestrial (*Luticola* spp., *Hantzschia amphioxys*, and *Stauroneis obtusa*), brackish–marine, and marine taxa (Fig. 7a).

The sedimentary structures recognized at location SZ1 provide information on current directions and flow conditions. The tsunami deposit records that this site was covered by three currents, which sequentially formed Units 1–3, Unit 4, and Unit 5. The climbing ripples with their leeside to seaward in Unit 3 (Fig. 4) indicate transport and deposition of sediments under a seaward current. Although the parallel and wavy laminations observed in basal parts of Units 1 and 2 cannot be used to reconstruct current direction, the structures in Units 1–3 likely formed sequentially under a single seaward current because no clear erosional boundaries separate these three units. The changes in mud content and the inverse grading of the sand fraction perhaps reflect an increase in the overall flow velocity during the formation of Unit 3 compared to Unit 2. In contrast, the change in sedimentary structures from parallel lamination in Unit 2 to climbing ripples in Unit 3 suggest a decrease in shear stress. These conflicting sedimentological features may be attributed to a specific environment at SZ1. SZ1 is located in the foot of a topographic step on the landward side, with a small seawall situated on the seaward side (Fig. 1e). In such an environment, the water depth at SZ1 might have increased over time, suggesting that Unit 3 was formed in a deeper environment than Unit 2. The preservation of the shape of the ripple trough depressions and the distinct boundary between Unit 3 and Unit 4 (visible as differences in radiodensity) suggest that Unit 4 was formed by a wave (or a current) different from the one that formed Units 1–3. Unit 4 is considered to be strongly related to the flat-head features at the top of Unit 3. As one of the hypotheses, immediately after deposition of Unit 3, the climbing ripples may have been redeposited in fluid because of a high porewater content; thus, the current that formed Unit 4 might make a shape like dragging the underlying sediment seaward, looking the surface being stretched and the wisp sticking into Unit 4. Continuation of dragging-like formation was likely to have caused flattening and possibly truncation, of the tops of the crests. The sharp lower contact of Unit 5 implies that another wave or current occurred after the phase that formed Unit 4. The differences in diatom assemblages in Unit 4 from those in Units 3 and 5 are consistent with Units 3, 4, and 5 having been formed during different sedimentary phases. A possible cause of the partial discontinuity of Unit 4 is erosion by the flow that formed Unit 5. Where a crest is heavily eroded, Unit 5 lies directly above Unit 3, with no Unit 4 remaining; otherwise, Unit 4 overlies the top crest of Unit 3. The presence of upward-fining in Unit 5 indicates decreasing flow velocity toward the final phase of deposition.

The sedimentary structures observed at SZ1 are probably specific to that location. When tsunami waves strike a seawall, reflections and scattering are generated, leading complicated flow patterns and sediment transportation and redeposition. Given such complex flows and a report of at least two tsunami waves running up this area^39^, it is not unreasonable to conclude that the complicated sedimentation of SZ1 was formed during different sedimentary phases under the three different currents. The specific conditions at this location resulted in difference in faces of the tsunami deposits between SZ1 and the other locations; the correlation of the tsunami deposits between SZ1 and the other locations is thus not established in this study.

#### Location SZ2

At location SZ2, located in a small cultivated field, the tsunami deposits are 4–6 cm thick and consist mainly of fine sand. The tsunami sand overlies a pre-tsunami organic soil (Fig. 5a). We divide this tsunami deposit into two parts based on a very faint change in sediment color in the field survey—the upper half browner, the lower part grayer. Mean grain size of the sand fraction in the SZ2 tsunami deposit is 2.33–2.66 phi, average 2.50 phi, and exhibits two slight upward-fining (Fig. 5a and Supplementary Data S2). The basal contact of the tsunami deposit is very clear both visually and in X-ray images; the tsunami sand fills irregularities on the pre-tsunami surface, and the underlying soil does not appear to have been eroded.

The upper and lower part of the tsunami deposit at SZ2 are correlated with those at SZ3 and SZ4, as mentioned below. However, we did not correlate the two units at this location with any part of the SZ1 succession because of the large difference in sedimentary features between SZ1 and the other locations.

#### Location SZ3

The tsunami deposit at location SZ3 within the cultivated field is 5–7 cm thick (Fig. 5b). Mean grain size of the sand fraction in the tsunami deposit at SZ3 is 2.39–2.78 phi (Fig. 5b and Supplementary Data S2), and is divided into an upper brown part (approximately 0–2 cm from the top of the deposit) and a lower gray part (approximately 2–7 cm) by visual observation in the field and laboratory. The CT and X-ray images of the tsunami deposit reveal parallel laminae in the upper part of the deposit (Fig. 5b). Grain-size analyses revealed two upward-fining in grain size within the tsunami deposit. The sand fraction of lowermost sample is poorly sorted (0.84 phi). The mean grain size of the sand fraction is still within the range of fine sand, but the lowest sample is coarse relative to upper ones at this location. The mud content of this sample (14.78 wt%) is the highest of all the tsunami deposit samples. The X-ray image shows that the basal contact with the underlying soil is distinct and the tsunami sand fill irregularities of the pre-tsunami ground surface. There is no clear evidence for erosion of the pre-tsunami ground surface (e.g., ripped mud clasts) on either the CT or X-ray images.

The diatom assemblages within the tsunami deposit are dominated by brackish–marine and marine benthic taxa (Fig. 7b). The brackish–marine species *Catenula adhaerens* exhibits the highest relative abundance in all tsunami deposit samples from this location. The pre-tsunami cultivated field soil is dominated by freshwater terrestrial taxa, such as *Luticola* spp. and *Hantzschia amphioxys*. *Tryblionella debilis* displays consistent abundance in the field soil.

The upper and lower parts of the tsunami deposit at SZ3 are correlated with those at SZ2 and SZ4, as mentioned below. No part of the tsunami deposit at this location correlates with any part of the tsunami deposits at SZ1 or SZ5.

#### Location SZ4

A 4–5-cm thick tsunami deposit was recognized at SZ4 in the cultivated field (Fig. 5c). Mean grain size of the sand fractions is 2.54–2.77 phi (Fig. 5c and Supplementary Data S2). Grain-size analyses showed two upward-fining in the tsunami deposit. Parallel- to cross-lamination is visible at 0–2 cm from the top on soft X-ray and CT images. The tsunami deposit is divided into an upper brown part (approximately 0–2 cm from the top of the deposit) and a lower dark gray part (approximately 2–5 cm) on the basis of difference in sediment color in the field survey, the difference in radiodensity, the boundary between the two upward-fining, and the presence/absence of parallel laminations in the laboratory analysis. The mud content decreases from bottom to top of the tsunami deposit, but is exceptionally high near the top of the deposit. The boundary between the tsunami deposit and the underlying soil is sharp.

The upper and lower parts at locations SZ2–SZ4 are correlated because the tsunami deposits at these locations share the characteristic that the boundary identified by a change in color coincides with the one defined by two upward-fining structures. The presence of two upward-fining structure at these three locations may suggest that each unit was formed by different two waves. This inference does not contradict the eyewitness report of at least two separate tsunami waves. However, the tsunami deposits at SZ2–SZ4 and those at SZ1 could not be correlated in this study. The difference in the pre-tsunami surface (SZ1 is on a paved footpath and SZ2–SZ4 are on cultivated field soil) and complicated water flow and sediment transportation around the seawall, as mentioned above, perhaps caused the variation in appearance of the tsunami deposits.

#### Location SZ5

At location SZ5, the most landward sampling location, the tsunami deposit is a 3–4-cm thick sand layer, exhibiting dark gray in color (Fig. 5d). Mean grain size of the sand fraction at the top is 3.00 phi, while mean grain size of the sand fraction in the other samples is 2.61–2.75 (Fig. 5d & Supplementary Data S2). Parallel laminations were observed on CT and X-ray images around the top of the deposit (approximately 0–2 cm below the top of the deposit).Grain-size analyses showed a single upward-fining in the sand fraction of the tsunami deposit (Fig. 5d and Supplementary Data S2). The mud content is highest at the top. The contact of the tsunami deposit with the underlying soil is distinct but not erosional. The tsunami deposit fills small-scale topographic irregularities of the pre-tsunami ground surface.

The diatom assemblages within the tsunami deposit at SZ5 are dominated by brackish–marine and marine species (Fig. 7c). The brackish–marine species *Catenula adhaerens* has high relative abundance in the lower part of the tsunami deposits. Toward the top, the abundances of the marine taxa *Delphineis* spp. and *Diploneis interrupta* increase to be greater than those of *C. dhaerens*. The pre-tsunami cultivated field soil is dominated by freshwater terrestrial taxa such as *Luticola* spp. and *Hantzschia amphioxys*, as also observed at SZ3.

Correlation of the tsunami deposits between SZ5 and the other locations is ambiguous. The tsunami deposit of SZ5 is difficult to divide into subunits, although parallel laminae are found around the top of the deposit. There is a single upward-fining of grain size at SZ5, whereas there are two gradings at the other locations.

### Sedimentology and diatom assemblages of vented sediments

Liquefaction-induced vented sediments were observed on paddy fields about 200–250 m from the post-tsunami shoreline. The diameters of the vented sediments range from 30 to 135 cm (Table 1). Grain-size and diatom analyses were carried out on the samples taken from the vented sediments at two locations, named SZ6 and SZ7 (Fig. 1c).

#### Location SZ6

A 11-cm thick brown vented sand with a diameter of 110 cm × 135 cm (Figs. 2j & 6a) was observed at SZ6 in the field. The sand fraction in the middle and lower parts consist mainly of fine sand (mean grain size of 2.03 phi) and (mean grain size of 1.84 phi), respectively (Supplementary Data S2). The mean grain size of both parts is coarser than that of any tsunami deposit samples (Table 2 and Supplementary Data S7). Gray organic paddy soil occurs at depths of 11–32 cm below the ground surface. Mean grain size of the sand fraction in paddy soil is 1.56 phi. The paddy soil has a higher mud content (25.80 wt%) than the vented sediments (3.10–4.99 wt%). A light brown sand layer underlying paddy soil was recognized below the paddy soil. The boundary of the vented sediments and the paddy soil and the one between the paddy soil and the underlying light brown sand were identified by means of differences in color. The basal surface of the sand underlying paddy soil was not observed in this study.

**Table 2:** The results of grain-size analysis of beach sand, and average grain-size values for tsunami deposits at SZ1–SZ5, cultivated field soil, vented sediments, paddy field soil, and the sand underlying paddy soil. Full results are provided in Supplementary Data S2.

Sample	Median [phi]	Mean [phi]	Sorting	Mud content [%]
Beach sand	2.39	2.38	0.45	5.20
Average of tsunami deposits	2.65	2.63	0.55	6.88
Average of cultivated field soil	1.78	1.73	0.76	9.90
Average of vented sediments	1.81	1.76	0.64	5.24
Average of paddy field soil	1.62	1.38	1.29	29.69
Average of the sand underlying paddy soil	1.56	1.47	0.85	11.76

The diatom assemblage in the middle part of the vented sediments at SZ6 is dominated by a freshwater benthic taxon, *Sellaphora* sp. 1 (Fig. 7d). The assemblages within the lower part of the vented sand and the paddy soil are characterized by the similar assemblages, including *Gomphonema* spp., *Pinnularia* spp., *Placoneis undulata*, *Sellaphora pupula* complex, and *Caloneis bacillum*. The sand underlying paddy soil contains fragments of marine plankton, *Thalassiosira* spp., but few individuals were observed as identifiable valves, and counting could not be carried out.

#### Location SZ7

A vented sand 55 cm in diameter and 14 cm thick was observed at SZ7 (Figs. 2k & 6b). The sand consists mainly of brown medium sand (Fig. 6b and Supplementary Data S2). Mean grain size of the sand fraction in the vented sediments is 1.60–1.67 phi. Organic paddy soil appears at depths of 14–30 cm below the surface. This paddy soil is muddy sand with the mud content of 28.53–33.55 wt%. Mean grain size of sand fraction consist mainly of coarse to medium sand (mean grain size is 0.69–1.72). Light brown medium sand below the paddy soil was recognized deeper than 30 cm from the top of the vented sand. The boundaries between layers were identified from difference in radio-density on CT and soft X-ray images and from differences in color.

The diatom assemblages within the lower part of the vented sand and the paddy soil are characterized by freshwater (*Gomphonema* spp., *Pinnularia* spp., *Placoneis undulata*, *Sellaphora pupula* complex) and freshwater–brackish (*Caloneis bacillum*) benthic taxa (Fig. 7e). The sand underlying paddy soil is dominated by marine plankton (*Thalassiosira* spp.). Samples from the upper and middle parts of the vented sediments have too few diatoms for counting.

### Tsunami sediment sources

There are several possibilities for the main sources of the tsunami deposits: the beach, the pre-tsunami ground surface, the riverbed of the Ukai River, and vented sediments. We estimated the main source of the tsunami sand based on the results of diatom and grain-size analyses. The comparable reference samples include vented sediments at SZ6 and SZ7, soil (pre-tsunami soil samples at SZ3, SZ5, SZ6, and SZ7), the sand below the paddy soil at from SZ7, and beach sand collected near our study site (Fig. 1c). The beach sand is well-sorted fine sand (mean grain size 2.38 phi) and is dominated by brackish–marine and marine taxa (*Catenula adhaerens*, Achnantheceae sp. 1, *Cocconeis* sp. 4, and *Delphineis* spp.) (Fig. 7f and Table 2; the result of grain-size analysis for the beach sand is provided in Supplementary Data S8). We could not recognize any fragments of sponge spicules or small shells in the beach sand sample under the binocular microscope.

**Fig. 8:**
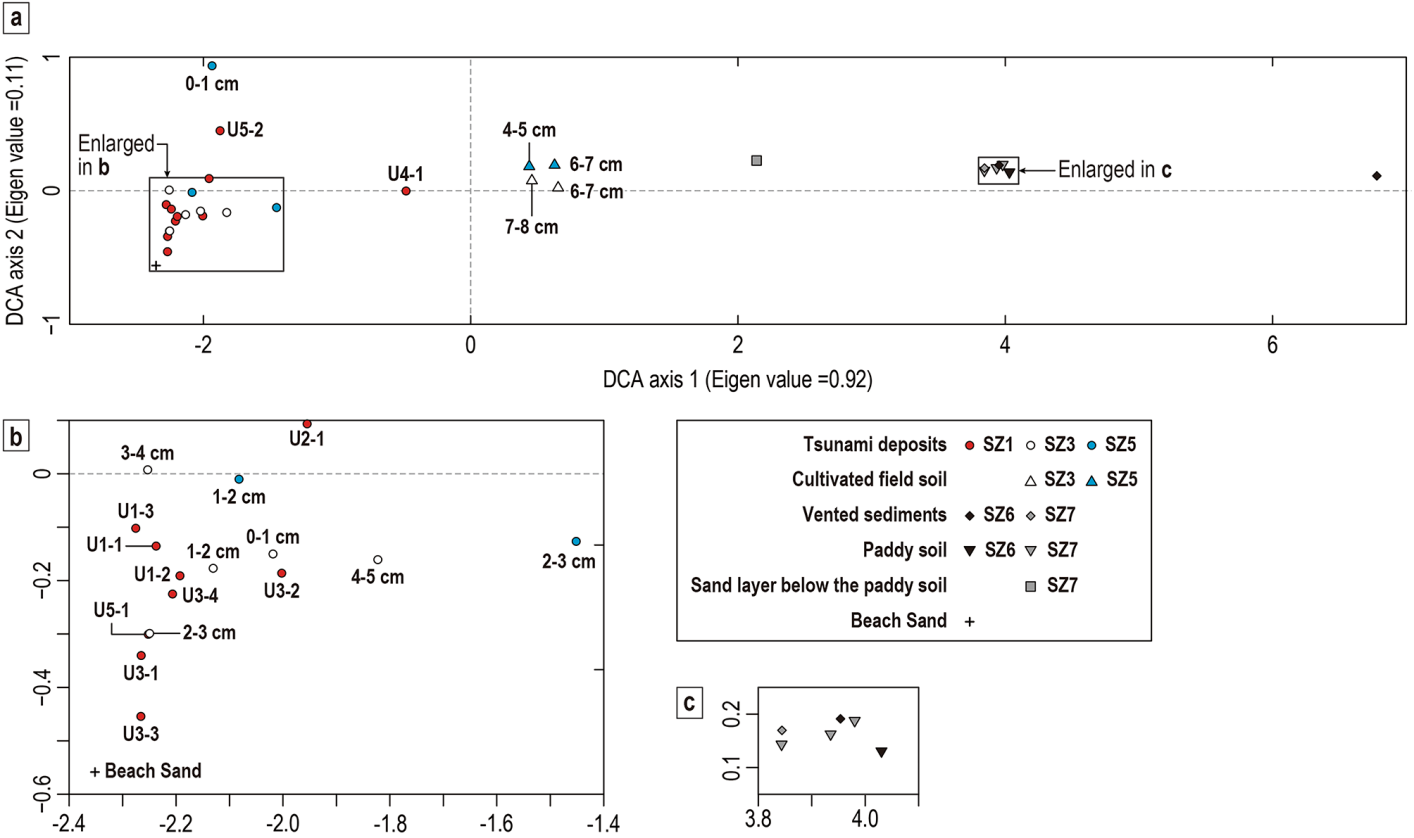
(**a**) Detrended correspondence analysis (DCA) results. The first and second DCA axes are used as the horizonal and vertical axes. (**b**) Enlarged view of the left rectangle in (**a**). (**c**) Enlarged view of the right rectangle in (**a**).

Detrended correspondence analysis (DCA) was carried out to understand the relationships of diatom assemblages among the tsunami deposits and reference samples (Fig. 8). The DCA ordination plots represent assemblage samples as points in multi-dimensional space. In the ordination, similar assemblages are located close together and dissimilar assemblages further apart^50^.

Most samples from the tsunami deposits at SZ1, SZ3, SZ5, and beach sand have similar positions in the DCA plot (Fig. 8), although a sample U4-1 from Unit 4 of SZ1 sits relatively close to those from the underlying field soil near the center of the ordination. This result suggests that a large portion of the tsunami deposits were eroded from or near the beach, transported, and deposited. The exceptional sample U4-1 of Unit 4 of SZ1 suggests that Unit 4 includes a different source from the other tsunami samples, perhaps some portion of cultivated field soil.

According to the DCA plots, the liquefaction-induced vented sediments are unlikely to be a major source of the tsunami deposits collected and analyzed in this study. On the DCA ordination plots, samples from the vented sand plot far from the tsunami deposits (Fig. 8). Paddy field soil and the underlying sand are also distant from the tsunami deposits in the DCA plots. We should note that our data only show reference samples of vented sediments from paddy fields, obtained about 120 m west of the sampled locations, because liquefied vented sediments were found to have contributed to the source of tsunami deposits in previous studies (e.g., the 2011 tsunami in Sendai, Japan^51^, and an event about 1100 years ago in Puget Sound, USA^52^). If vented sediments that have different characteristics from those at SZ6 and SZ7 had appeared on the surface near our transect before the tsunami inundation, they could have been the origin of the tsunami deposit, although we are not aware of any eyewitness accounts of liquefaction prior to the tsunami arrival.

The results of the grain-size analysis support the estimates from diatom analysis. The mean grain sizes of the sand fractions in the tsunami deposits at locations SZ1–SZ5 are largely fine (2.16–2.84 on the phi scale), except for very fine at the top of SZ5 (3.00 phi) (Figs. 4 and 5). In contrast, the mean grain sizes of the sand fractions of paddy field soil (0.69–1.72 phi), sand underlying paddy field soil (1.43–1.51 phi), and cultivated field soil (1.51–1.84 phi) are coarse relative to the tsunami sand. Four of the five samples from the vented sediments also show coarser grain sizes (1.60–1.84 phi) than those of the tsunami deposits (Table 2, Supplementary Data S2). Only the beach sample has a similar grain size (2.38 phi) to the tsunami deposits among the comparative reference samples (Table 2, and Supplementary Data S2).

The sediment source for Unit 4 of SZ1 remains uncertain relative to other sedimentary units at this and other locations. As described earlier, Unit 4 is characterized by a mixed diatom assemblage that includes freshwater terrestrial, brackish–marine, and marine taxa. On the DCA ordination, the sample from this unit is positioned closer to cultivated soil samples than to the beach sample, suggesting that this unit may have been transported from freshwater environments. However, the mean grain size of the sand fraction of Unit 4 (2.16 phi) is finer than that of our comparative reference samples from freshwater environments (Table 2). Although the mean grain size of Unit 4 is similar to that of the beach reference sample, the beach sample lacks the sponge spicules and shell fragments that dominate Unit 4. It is currently unclear whether our beach sand sample unexpectedly did not contain sponge spicules and shell fragments immediately after severe erosion of beach by the tsunami, or whether the sponge spicules and shell fragments were sourced from different environments, such as the deeper sea bottom.

## Conclusion

Case studies describing modern tsunami deposits have increased since the 2004 Sumatra and 2011 Tohoku tsunamis, but their number remains small compared to paleo-tsunami studies. In this study, we carried out a field survey in the disaster area within a month of the 2024 Noto Penindula earthquake to document modern tsunami deposits. Our detailed observations of the tsunami deposits using soft X-ray and X-ray CT images and grain-size analysis revealed that the changes in sedimentary structures and grain-size within each sampled location as well as among them. There was a remarkable difference in sedimentary structures between the most seaward location SZ1 and others, even within a small area of about 50 m from the shoreline. The intricate tsunami deposits, found only near the seawall (SZ1), were likely formed by at least three currents. Diatom assemblages indicated that, although some sediment sources include cultivated field soils and possibly and deeper-marine sediments, most are composed of beach sand. The deposition of these tsunami sediments, influenced predominantly by seaward currents rather than landward ones despite their primarily marine origin, was likely due to rapid changes in current velocity and direction caused by the presence of a seawall, which behaved as an obstacle. In more landward locations (SZ2–SZ5), the tsunami deposits are generally massive, with faint laminae and slight grading in grain size. This geological archive, including photographs, X-ray images, grain-size data, and diatom assemblages, provides a valuable analogue for comparing paleo-tsunami deposits and informing coastal development considerations.

## Methods

### Field survey

Fieldwork was carried out in January, February, and June 2024. The thickness of the tsunami deposit was measured at 0.5-m intervals at 96 locations up to 48 m from the seawall. For detailed observation of sedimentary structures and paleontological analysis, sediment samples were collected at five locations (SZ1–SZ5) by pushing a flat acrylic box into the wall of a small pit. Location SZ1 was on a path by the seawall; locations SZ2, 3, 4, and 5 were in a small cultivated field landward of the path. The locations for sample collection and observations and the heights of debris and watermarks were measured with a network Real Time Kinematic-Global Navigation Satellite System (RTK-GNSS) survey system from Leica Geosystems Inc. (Norcross, Georgia, USA).

### Observation of sediments

We observed the sediment samples visually, and also obtained soft X-ray and X-ray computed tomography (CT) images. Soft X-ray and X-ray CT images were taken by using a SOFRON SRO-i503-2 (SOFTEX Co., Ltd., Tokyo, Japan) and with a Hitachi Supria Grande PREMIUM (Hitachi, Ltd., Tokyo, Japan), respectively, at the Geological Survey of Japan. Observation of the CT images and creation of a 3D movie were performed using the software OsiriX MD (Pixmeo SARL, Bernex, Switzerland)^53^.

### Grain-size analysis

Grain-size analysis was conducted on samples from locations SZ1–SZ5, including the tsunami deposit and underlying field soil at the study site. The sampling interval was basically every 1 cm. As Unit 4 of SZ1 is not stratified, the samples from Unit 4 and its adjacent layers were taken without crossing the boundaries of each unit (Supplementary Data S5). The vented sediments and paddy field soil at SZ6 and SZ7 and the light brown sand underlying paddy soil at SZ7 were also included in the grain-size analysis. As a reference sample, the grain size of beach sand (Fig. 1c) was measured. Bulk samples were sieved using mesh cloth with 63 μm (4 phi) openings to measure the mud content. The grain-size distribution of sand particles was measured using an image analyzer (Camsizer, Retsch Technology GmbH, Haan, Germany). The mean, median, sorting, skewness, and kurtosis of the grain-size distributions were calculated using the logarithmic graphical method^54^. The detailed results are shown in Supplementary Data S2.

### Diatom analysis

Diatom assemblages were identified and counted within the tsunami deposits at SZ1, SZ3, and SZ5 as well as in underlying cultivated field soil at SZ3 and SZ5, beach sand (Fig. 1c), vented sediments at SZ6 and SZ7, field soil at SZ6 and SZ7, and the light brown sand underlying paddy soil at SZ7. For tsunami deposits, subsampling was conducted at 1-cm intervals. As Unit 4 at SZ1 is not stratified, the samples from Unit 4 and its adjacent layers were taken without crossing the boundaries of each unit (Supplementary Data S5). Slides were prepared with a bleaching method^55,56^. For each slide, at least 200 diatom valves were identified under a light microscope at × 600 magnification. Diatom identification and ecological information followed standard and local studies^57–66^. Count data are provided in Supplementary Data S6. Detrended correspondence analysis (DCA) was performed with “vegan” (version 2.5-3)^67^, a package written for the free open-source language R (version 4.4.1)^68^. The script is provided in Supplementary Data S9.

## Supplementary Information

Caption of Supplementary Data S4.

Supplementary Data S1.

Supplementary Data S2.

Supplementary Data S3.

Supplementary Data S4.

Supplementary Data S5.

Supplementary Data S6.

Supplementary Data S7.

Supplementary Data S8.

Supplementary Data S9.

## Data Availability

All data integral to the stated conclusions are presented within the paper and Supplementary Data.
