# Supplementary material for: Sedimentological and micropaleontological characteristics of tsunami deposits associated with the 2024 Noto Peninsula earthquake
Source: Sci Rep. 2025 Mar 21;15:9820. doi: 10.1038/s41598-025-90945-w (PMC11928632; doi:10.1038/s41598-025-90945-w)
Supplement: Supplementary file 1 — Caption of Supplementary Data S4. [file 41598_2025_90945_MOESM1_ESM.pdf]

Supplementary Data S4. A 3D image of the tsunami deposits at SZ1 constructed by OsiriX[55].
