# Supplementary figures and images for: Sedimentological and micropaleontological characteristics of tsunami deposits associated with the 2024 Noto Peninsula earthquake
Source: Sci Rep. 2025 Mar 21;15:9820. doi: 10.1038/s41598-025-90945-w (PMC11928632; doi:10.1038/s41598-025-90945-w)

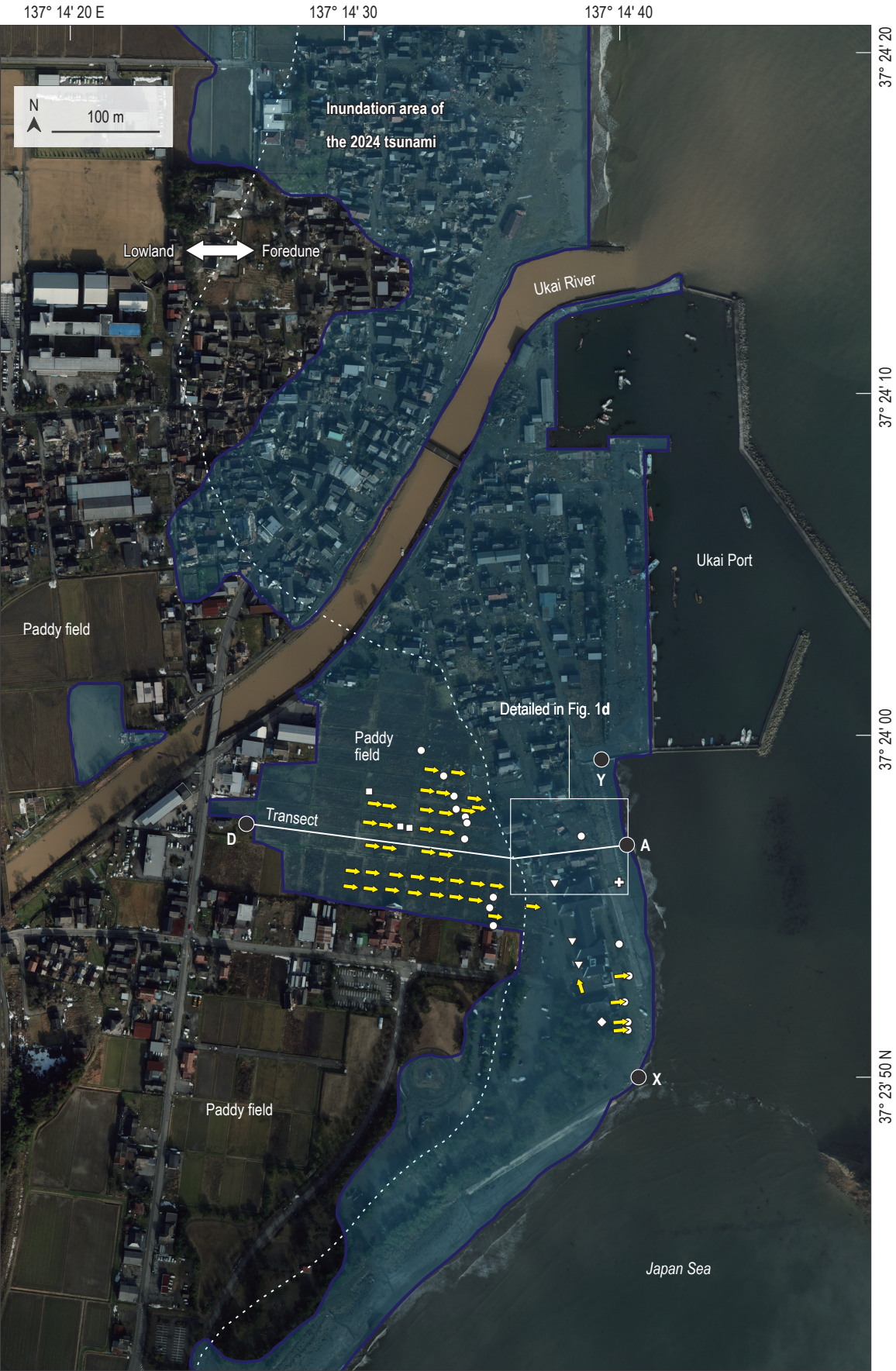

- Debris
- ▽ Water marks
- ◇ Tsunami deposit in the park
- Vented sediments
- ⊕ Beach sand sample

Supplement: Supplementary file 2 — Supplementary Data S1. [file 41598_2025_90945_MOESM2_ESM.pdf]

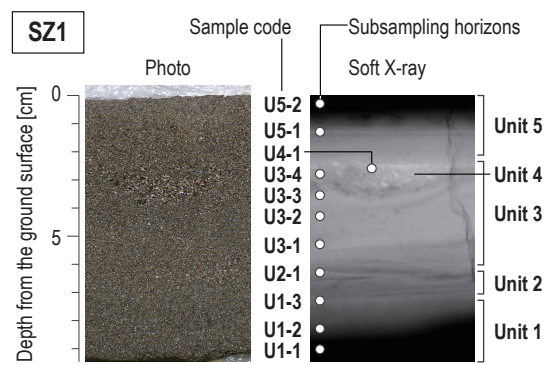

Supplement: Supplementary file 6 — Supplementary Data S5. [file 41598_2025_90945_MOESM6_ESM.pdf]

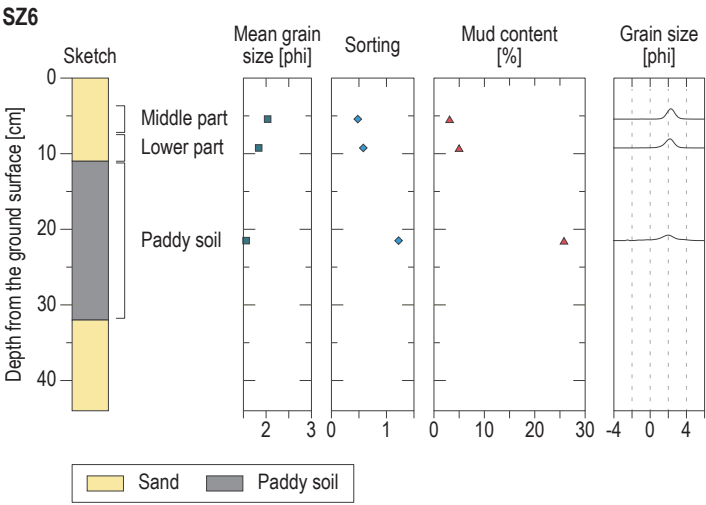

Supplement: Supplementary file 8 — Supplementary Data S7. [file 41598_2025_90945_MOESM8_ESM.pdf]

Beach sand : sampled at the point of cross mark in Fig. 1c. on 19th January 2024.

Result of grain-size analysis

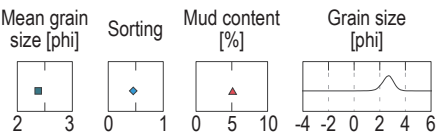

Supplement: Supplementary file 9 — Supplementary Data S8. [file 41598_2025_90945_MOESM9_ESM.pdf]
