# Supplementary material for: Sedimentological and micropaleontological characteristics of tsunami deposits associated with the 2024 Noto Peninsula earthquake
Source: Sci Rep. 2025 Mar 21;15:9820. doi: 10.1038/s41598-025-90945-w (PMC11928632; doi:10.1038/s41598-025-90945-w)
Supplement: Supplementary file 10 — Supplementary Data S9. [file 41598_2025_90945_MOESM10_ESM.pdf]

Supplementary Data S9. The script for the detrended correspondence analysis (DCA) in the R Studio environment.

```
> install.packages("vegan")
> library(vegan)
> suzu1022 <- read.csv("~/suzu1022.csv", row.names=NULL)
> print(suzu1022)
                       X Achnantheceae.sp..1 Amphora.proschkiniana Caloneis.bacillum Catenula.adhaerens Cocconeis.sp..1
1                SZ1 U5-2           0.4608295             1.3824885         0.0000000           7.834101       0.9216590
2                SZ1 U5-1           3.3980583             1.9417476         0.0000000          16.019417       1.9417476
3                SZ1 U4-1           0.4854369             0.9708738         0.0000000           8.252427       0.9708738
4                SZ1 U3-4           2.4875622             1.9900498         0.0000000          20.398010       6.9651741
5                SZ1 U3-3           1.4851485             2.9702970         0.0000000          28.217822       2.9702970
6                SZ1 U3-2           2.9702970             3.4653465         0.0000000          16.831683       2.9702970
7                SZ1 U3-1           2.9268293             3.4146341         0.0000000          20.975610       2.4390244
8                SZ1 U2-1           2.4752475             1.9801980         0.0000000          15.346535       1.4851485
9                SZ1 U1-3           1.4705882             3.4313725         0.0000000          14.705882       2.9411765
10               SZ1 U1-2           2.4630542             0.0000000         0.0000000          20.197044       3.4482759
11               SZ1 U1-1           3.3980583             0.9708738         0.0000000          19.417476       2.4271845
12             SZ3 0-1 cm           4.4776119             2.4875622         0.0000000          12.437811       1.9900498
13             SZ3 1-2 cm           3.7209302             1.3953488         0.0000000          14.883721       2.3255814
14             SZ3 2-3 cm           4.8543689             1.4563107         0.0000000          19.417476       0.0000000
15             SZ3 3-4 cm           1.3698630             3.1963470         0.0000000          10.958904       0.0000000
16             SZ3 4-5 cm           3.9603960             3.9603960         0.0000000          17.326733       0.4950495
17             SZ3 6-7 cm           0.0000000             0.0000000         1.4563107           0.000000       0.0000000
18             SZ3 7-8 cm           0.0000000             0.0000000         0.0000000           0.000000       0.0000000
19             SZ5 0-1 cm           0.0000000             1.9801980         0.4950495           5.940594       2.4752475
20             SZ5 1-2 cm           2.4271845             1.9417476         0.0000000          12.621359       0.0000000
21             SZ5 2-3 cm           1.9512195             0.9756098         0.0000000          17.560976       0.4878049
22             SZ5 4-5 cm           0.0000000             0.0000000         0.9900990           0.990099       0.0000000
23             SZ5 6-7 cm           0.0000000             0.0000000         0.0000000           0.000000       0.0000000
24 SZ6 Vented sediment 1            0.0000000             0.0000000         0.0000000           0.000000       0.0000000
25 SZ6 Vented sediment 2            0.0000000             0.0000000         4.9019608           0.000000       0.0000000
26              SZ6 Paddy           0.0000000             0.0000000         6.0000000           0.000000       0.0000000
27 SZ7 Vented sediment 1            0.0000000             0.0000000         7.7669903           0.000000       0.0000000
28             SZ7 Paddy1           0.0000000             0.0000000        11.4285714           0.000000       0.0000000
29             SZ7 Paddy2           0.0000000             0.0000000         5.7142857           0.000000       0.0000000
30             SZ7 Paddy3           0.0000000             0.0000000         7.1428571           0.000000       0.0000000
31         SZ7 Sand layer           0.0000000             0.0000000         2.5000000           0.000000       0.0000000
32              BeachSand          10.3448276             5.9113300         0.0000000          12.315271       1.4778325
   Cocconeis.sp..2 Cocconeis.sp..3 Cocconeis.sp..4 Cocconeis.sp..5 Delphineis.spp. Diploneis.interrupta Eunotia.spp.
1        8.2949309       0.4608295       0.4608295       0.0000000      11.5207373            0.0000000    0.0000000
```

2	1.9417476	4.8543689	1.9417476	0.4854369	6.3106796	0.0000000	0.0000000
3	0.9708738	0.4854369	0.0000000	0.9708738	3.8834951	0.0000000	0.0000000
4	3.4825871	1.9900498	3.9800995	1.4925373	7.4626866	0.0000000	0.0000000
5	2.9702970	0.9900990	5.4455446	1.4851485	5.9405941	0.0000000	0.0000000
6	4.4554455	3.4653465	1.9801980	1.4851485	6.4356436	0.0000000	0.0000000
7	3.9024390	1.4634146	5.3658537	0.4878049	7.8048780	0.0000000	0.0000000
8	5.9405941	0.0000000	0.0000000	0.0000000	12.8712871	0.0000000	0.0000000
9	3.9215686	6.3725490	2.4509804	0.4901961	7.3529412	0.0000000	0.0000000
10	1.4778325	3.9408867	2.9556650	0.9852217	10.3448276	0.0000000	0.0000000
11	4.8543689	3.8834951	3.3980583	0.0000000	11.1650485	0.0000000	0.0000000
12	5.9701493	1.4925373	4.4776119	0.0000000	8.4577114	0.0000000	0.0000000
13	3.2558140	2.3255814	6.0465116	0.4651163	10.2325581	0.0000000	0.0000000
14	5.3398058	3.8834951	5.3398058	1.4563107	8.2524272	0.0000000	0.0000000
15	4.1095890	1.8264840	5.0228310	5.4794521	9.5890411	0.0000000	0.0000000
16	5.9405941	1.4851485	2.9702970	0.9900990	8.9108911	0.0000000	0.0000000
17	0.0000000	0.0000000	0.0000000	0.0000000	0.0000000	0.0000000	0.0000000
18	0.0000000	0.0000000	0.0000000	0.0000000	0.0000000	0.0000000	0.0000000
19	6.4356436	2.4752475	0.9900990	0.9900990	17.3267327	6.9306931	0.0000000
20	2.4271845	1.9417476	5.8252427	0.9708738	12.6213592	0.9708738	0.0000000
21	2.9268293	0.0000000	2.4390244	0.4878049	6.8292683	0.0000000	0.0000000
22	0.0000000	0.0000000	0.0000000	0.0000000	0.0000000	0.0000000	0.0000000
23	0.0000000	0.0000000	0.0000000	0.0000000	0.0000000	0.0000000	0.0000000
24	0.0000000	0.0000000	0.0000000	0.0000000	0.0000000	0.0000000	0.0000000
25	0.0000000	0.0000000	0.0000000	0.0000000	0.4901961	0.0000000	5.8823529
26	0.0000000	0.0000000	0.0000000	0.0000000	0.0000000	0.0000000	0.0000000
27	0.0000000	0.0000000	0.0000000	0.0000000	0.4854369	0.0000000	0.4854369
28	0.0000000	0.0000000	0.0000000	0.0000000	0.0000000	0.0000000	0.0000000
29	0.0000000	0.0000000	0.0000000	0.0000000	0.0000000	0.0000000	0.0000000
30	0.0000000	0.0000000	0.0000000	0.0000000	0.0000000	0.0000000	0.4464286
31	0.0000000	0.0000000	0.0000000	0.0000000	0.0000000	0.0000000	0.0000000
32	2.4630542	3.4482759	10.3448276	0.9852217	6.8965517	0.0000000	0.0000000

	Gomphonema.spp.	Hanzschia.amphyoxis	Luticola.spp.	Stauroneis.obtusa	Sellaphora.sp..1	Pinnularia.borealis.complex
1	0.000000	0.4608295	1.8433180	0.0000000	0.0000000	0.9216590
2	0.000000	0.0000000	0.0000000	0.0000000	0.0000000	0.0000000
3	0.000000	6.3106796	17.4757282	5.3398058	0.0000000	1.4563107
4	0.000000	0.0000000	0.0000000	0.0000000	0.0000000	0.9950249
5	0.000000	0.0000000	0.4950495	0.0000000	0.0000000	0.0000000
6	0.000000	0.4950495	1.4851485	0.4950495	0.0000000	0.0000000
7	0.000000	0.0000000	0.4878049	0.0000000	0.0000000	0.0000000
8	0.000000	0.0000000	1.4851485	0.4950495	0.0000000	0.0000000
9	0.000000	0.0000000	0.4901961	0.0000000	0.0000000	0.0000000
10	0.000000	0.0000000	0.0000000	0.0000000	0.0000000	0.0000000
11	0.000000	0.0000000	0.0000000	0.0000000	0.0000000	0.0000000

12	0.000000	0.0000000	0.4975124	0.4975124	0.0000000	0.0000000
13	0.000000	0.9302326	0.4651163	0.0000000	0.0000000	0.0000000
14	0.000000	0.0000000	0.9708738	0.0000000	0.0000000	0.0000000
15	0.000000	0.0000000	0.4566210	0.0000000	0.0000000	0.0000000
16	0.000000	1.4851485	4.4554455	1.9801980	0.0000000	0.0000000
17	0.000000	10.1941748	35.4368932	4.8543689	0.0000000	1.9417476
18	0.000000	13.4259259	30.5555556	8.3333333	0.0000000	5.0925926
19	0.000000	2.4752475	0.4950495	0.0000000	0.0000000	0.0000000
20	0.000000	0.0000000	0.0000000	0.0000000	0.0000000	0.4854369
21	0.000000	5.8536585	5.3658537	0.4878049	0.0000000	0.4878049
22	0.000000	19.8019802	28.2178218	5.9405941	0.0000000	5.4455446
23	0.000000	21.7391304	32.8502415	5.3140097	0.0000000	3.3816425
24	0.000000	0.0000000	0.0000000	0.0000000	64.9038462	0.0000000
25	12.254902	1.4705882	0.0000000	0.0000000	3.9215686	0.0000000
26	14.500000	0.0000000	0.0000000	0.0000000	0.0000000	0.0000000
27	11.650485	0.0000000	0.0000000	0.0000000	0.0000000	0.0000000
28	16.666667	0.0000000	0.0000000	0.0000000	0.4761905	0.0000000
29	8.095238	0.0000000	0.0000000	0.4761905	0.0000000	0.0000000
30	8.482143	0.0000000	0.4464286	0.0000000	0.0000000	0.0000000
31	1.500000	0.0000000	0.0000000	0.0000000	0.0000000	0.0000000
32	0.000000	0.0000000	0.0000000	0.0000000	0.0000000	0.0000000

	Pinnularia.spp.	Placoneis.undulata	Sellaphora.pupula.complex	Stauroneis.sp..1	Thalassiosira.spp.	Tryblionellla.debilis
1	0.0000000	0.0000000	0.0000000	0.0000000	0.9216590	0.0000000
2	0.0000000	0.0000000	0.0000000	0.0000000	0.4854369	0.0000000
3	1.4563107	0.0000000	0.0000000	0.0000000	0.0000000	0.4854369
4	0.0000000	0.0000000	0.0000000	0.0000000	0.4975124	0.0000000
5	0.0000000	0.0000000	0.0000000	0.0000000	0.0000000	0.0000000
6	1.4851485	0.0000000	0.0000000	0.0000000	0.0000000	0.0000000
7	0.0000000	0.0000000	0.0000000	0.0000000	0.0000000	0.0000000
8	0.4950495	0.0000000	0.0000000	0.0000000	0.9900990	0.0000000
9	0.0000000	0.0000000	0.0000000	0.0000000	0.0000000	0.0000000
10	0.0000000	0.0000000	0.0000000	0.0000000	0.9852217	0.0000000
11	0.0000000	0.0000000	0.0000000	0.0000000	0.4854369	0.0000000
12	0.0000000	0.4975124	0.0000000	0.0000000	1.4925373	0.0000000
13	0.0000000	0.0000000	0.0000000	0.0000000	0.9302326	0.0000000
14	0.0000000	0.0000000	0.0000000	0.0000000	0.0000000	0.0000000
15	0.0000000	0.0000000	0.0000000	0.0000000	0.0000000	0.0000000
16	0.0000000	0.0000000	0.0000000	0.0000000	0.9900990	0.0000000
17	5.8252427	0.0000000	0.0000000	0.0000000	0.0000000	12.1359223
18	2.7777778	0.0000000	0.0000000	0.0000000	0.0000000	6.0185185
19	0.0000000	0.0000000	0.0000000	0.0000000	0.4950495	0.0000000
20	0.9708738	0.0000000	0.0000000	0.0000000	0.4854369	0.0000000
21	0.9756098	0.0000000	0.0000000	0.0000000	0.0000000	0.4878049

```
22    1.9801980   0.0000000   0.0000000  0.0000000   0.0000000  3.9603960
23    7.7294686   0.4830918   0.0000000  0.0000000   0.0000000  2.8985507
24    1.4423077   0.4807692   0.4807692  6.7307692   0.0000000  0.0000000
25   13.2352941   6.8627451   3.4313725  0.9803922   0.0000000  0.0000000
26    7.0000000  15.0000000   9.5000000  0.5000000   0.0000000  0.0000000
27    7.2815534  10.6796117   9.7087379  0.0000000   1.4563107  0.0000000
28    7.6190476   7.1428571  10.0000000  0.0000000   0.0000000  0.0000000
29    6.6666667  11.4285714   5.2380952  0.0000000   0.4761905  0.0000000
30    5.3571429  10.7142857   7.1428571  0.0000000   0.0000000  0.0000000
31    2.0000000   1.0000000   0.0000000  0.0000000  49.5000000  0.0000000
32    0.0000000   0.0000000   0.0000000  0.0000000   0.0000000  0.0000000
> dcadata=suzu1022[,c(-1)]
> dca=decorana(dcadata, ira=0)
> summary(dca)

Call:
decorana(veg = dcadata, ira = 0)

Detrended correspondence analysis with 26 segments.
Rescaling of axes with 4 iterations.
Total inertia (scaled Chi-square): 3.7819

                      DCA1    DCA2    DCA3    DCA4
Eigenvalues         0.9248 0.10649 0.35947 0.21969
Additive Eigenvalues 0.9248 0.08897 0.36348 0.04303
Decorana values     0.9330 0.13523 0.09021 0.04643
Axis lengths        9.1343 1.49640 2.29976 1.81910

Species scores:

                           DCA1    DCA2    DCA3    DCA4 Totals
Achnantheceae.sp..1     -2.4327 -1.7117 -0.1637 -0.3501  57.13
Amphora.proschkiniana   -2.3425 -0.5062  0.0460  0.6190  45.82
Caloneis.bacillum        3.8163  0.5736 -1.4330 -1.2538  48.40
Catenula.adhaerens      -2.2628 -0.9641  0.0902 -0.3424 312.65
Cocconeis.sp..1         -2.3831  0.3410 -0.1753 -0.9596  38.73
Cocconeis.sp..2         -2.2309  1.1477  0.0739  0.1586  81.08
Cocconeis.sp..3         -2.5325  0.2037 -0.3545 -0.8236  46.78
Cocconeis.sp..4         -2.5336 -1.0953 -0.3908  0.5999  71.44
Cocconeis.sp..5         -2.3127  0.4951  0.1619  1.3207  19.71
Delphineis.spp.         -2.0949  1.3058  0.4306  0.5061 181.18
Diploneis.interrupta    -2.0359  2.8242 -0.5546 -0.5521   7.90
Eunotia.spp.             4.5938  0.3400  4.3258  3.7781   6.81
```

Gomphonema.spp.	4.2578	0.0998	-0.5962	0.1424	73.15
Hanzschia.amphyoxis	0.4121	0.9689	0.1095	0.9035	84.64
Luticola.spp.	0.3148	-0.1509	0.2063	-0.3690	163.98
Stauroneis.obtusa	0.2948	-0.3094	-0.3831	1.1587	34.21
Sellaphora.sp..1	6.9052	0.1127	1.4402	1.1863	69.30
Pinnularia.borealis.complex	0.1898	0.1623	-0.9863	1.2153	20.21
Pinnularia.spp.	2.7385	0.0816	1.5092	1.1803	74.30
Placoneis.undulata	4.2023	0.0343	-1.5994	-1.4226	64.29
Sellaphora.pupula.complex	4.3538	0.0721	-1.7167	-1.4889	45.50
Stauroneis.sp..1	6.7974	0.1132	1.6536	1.3888	8.21
Thalassiosira.spp.	1.9244	0.2218	-0.9208	-0.6781	60.19
Tryblionellla.debilis	0.6881	-0.1838	1.2235	-1.2170	25.99

Site scores:

	DCA1	DCA2	DCA3	DCA4	Totals
[1,]	-1.87520	0.44944	0.12503	0.12407	35.5
[2,]	-2.25444	-0.30273	0.01654	-0.16163	39.3
[3,]	-0.48358	-0.00183	0.11866	0.16184	49.5
[4,]	-2.20909	-0.22726	0.00609	-0.10410	51.7
[5,]	-2.26837	-0.45638	0.04831	-0.06580	53.0
[6,]	-2.00488	-0.18827	0.09208	-0.02131	48.0
[7,]	-2.26774	-0.34243	0.04781	-0.02814	49.3
[8,]	-1.95731	0.09133	0.15479	0.02467	43.6
[9,]	-2.27796	-0.10419	0.02630	-0.11957	43.6
[10,]	-2.19521	-0.19311	0.04440	-0.13796	46.8
[11,]	-2.23986	-0.13749	0.05658	-0.09269	50.0
[12,]	-2.02121	-0.15236	-0.00669	-0.01873	44.8
[13,]	-2.13311	-0.17915	0.02695	0.00623	47.0
[14,]	-2.25199	-0.30108	0.03814	-0.01673	51.0
[15,]	-2.25557	0.00533	0.08843	0.28158	42.0
[16,]	-1.82514	-0.16294	0.05770	0.05999	55.0
[17,]	0.65442	0.03374	0.36476	-0.07798	71.8
[18,]	0.45803	0.08712	0.16790	0.19113	66.2
[19,]	-1.93506	0.93579	0.04826	0.07556	49.5
[20,]	-2.08476	-0.01228	0.08326	0.15683	43.7
[21,]	-1.45256	-0.12620	0.14066	0.09495	47.3
[22,]	0.43837	0.19342	0.10172	0.25126	67.3
[23,]	0.62773	0.20333	0.24499	0.30504	74.4
[24,]	6.78010	0.11137	1.42066	1.17024	74.0
[25,]	3.95377	0.19119	0.40918	0.48965	53.4
[26,]	4.03047	0.12792	-0.87909	-0.60925	52.5
[27,]	3.84435	0.16965	-0.80517	-0.56632	49.5

[28,]	3.98035	0.18488	-0.80102	-0.51468	53.3
[29,]	3.84354	0.14063	-0.80972	-0.57676	38.1
[30,]	3.93534	0.15976	-0.87042	-0.64886	39.7
[31,]	2.13917	0.22585	-0.86083	-0.62915	56.5
[32,]	-2.35416	-0.56061	-0.04657	0.05444	54.2
