# Supplementary material for: Sedimentological and micropaleontological characteristics of tsunami deposits associated with the 2024 Noto Peninsula earthquake
Source: Sci Rep. 2025 Mar 21;15:9820. doi: 10.1038/s41598-025-90945-w (PMC11928632; doi:10.1038/s41598-025-90945-w)

Shimada et al. Supplementary Data S3

Photographs, sketches, soft X-ray images, and CT images of the tsunami deposits. (a) at SZ1, (b) trench photograph at SZ1, (c) at SZ2, (d) at SZ3, (e) at SZ4, (f) at SZ5. Note that the sea direction is to the right in all images except the photograph of the trench, in which the sea is to the left of the image.

(a) Location: SZ1

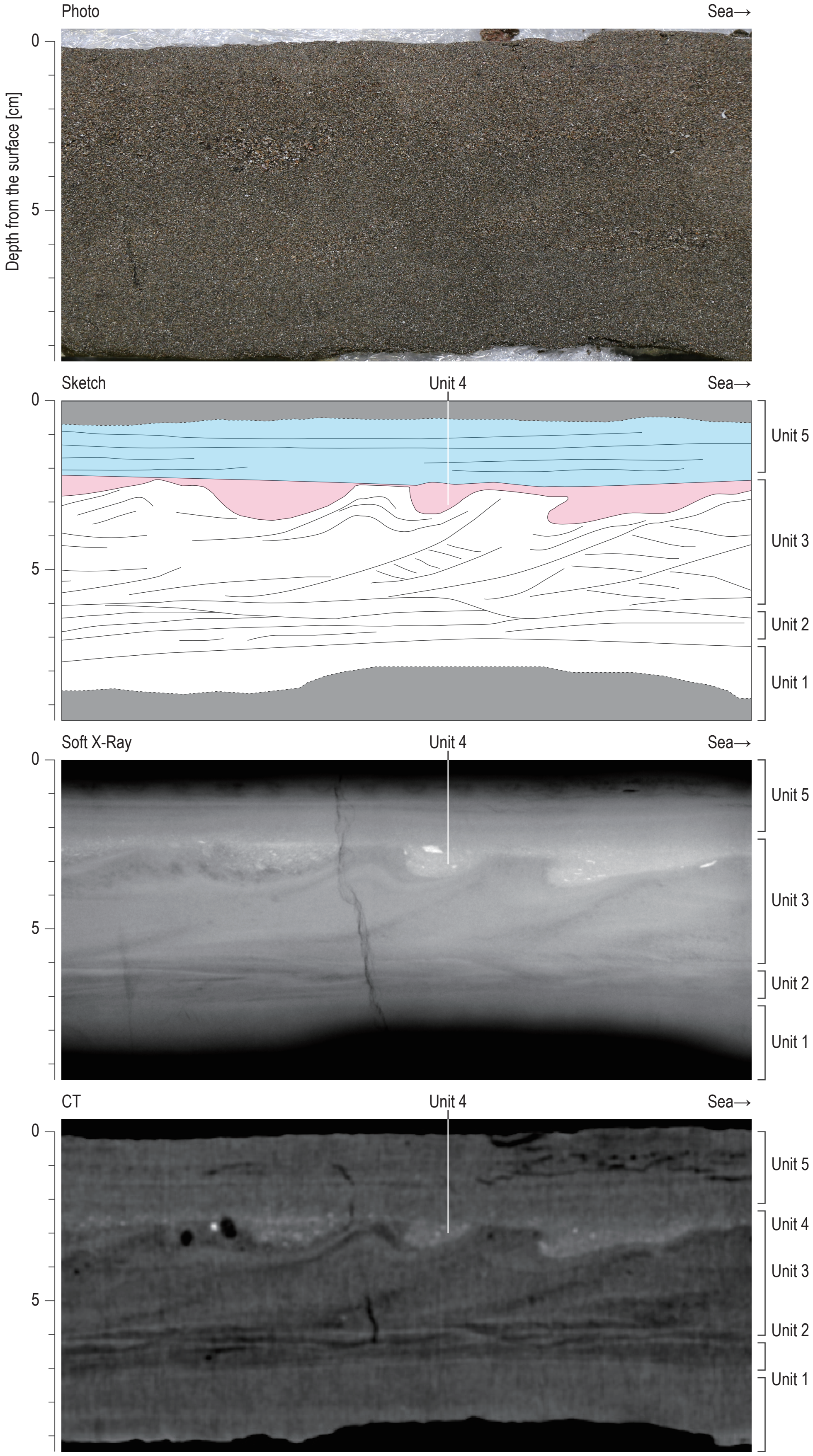

Shimada et al. Supplementary Data S3 ***Continued***
Photographs, sketches, soft X-ray images, and CT images of the tsunami deposits. (a) at SZ1, (b) trench photograph at SZ1, (c) at SZ2, (d) at SZ3, (e) at SZ4, (f) at SZ5. Note that the sea direction is to the right in all images except the photograph of the trench, in which the sea is to the left of the image.

(b) Location: SZ1

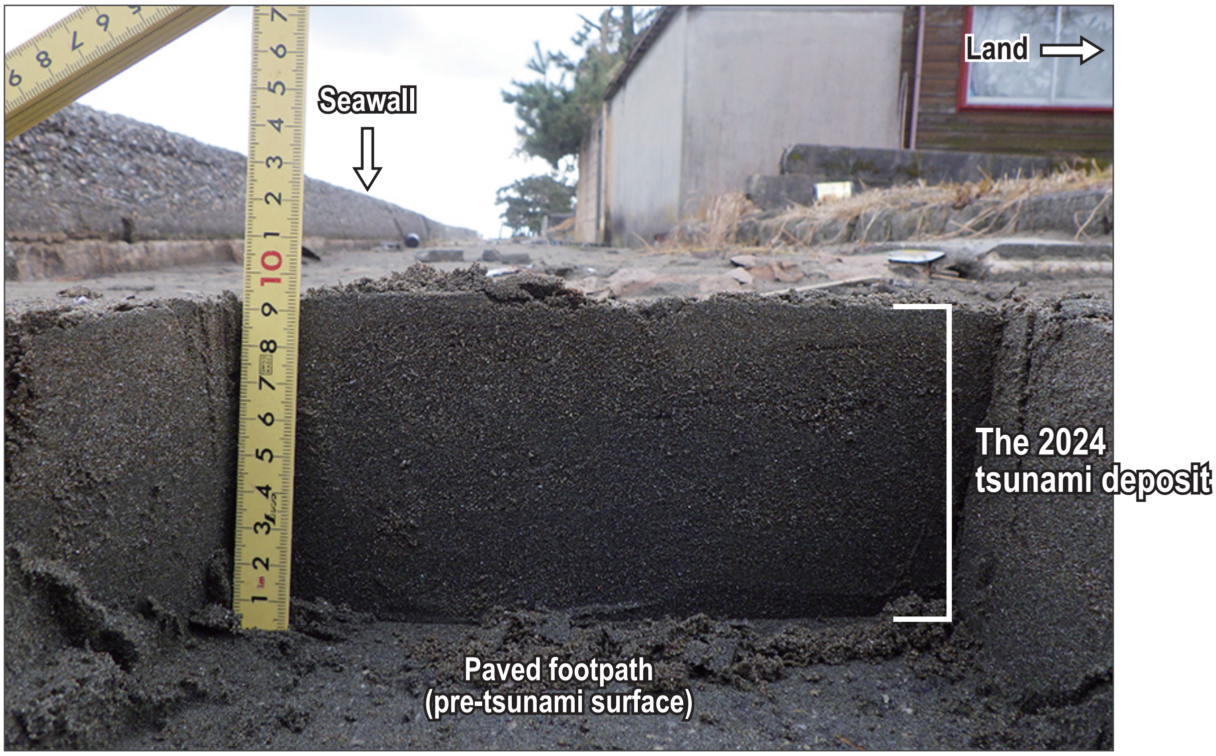

Shimada et al. Supplementary Data S3 *Continued*
Photographs, sketches, soft X-ray images, and CT images of the tsunami deposits. (a) at SZ1, (b) trench photograph at SZ1, (c) at SZ2, (d) at SZ3, (e) at SZ4, (f) at SZ5. Note that the sea direction is to the right in all images except the photograph of the trench, in which the sea is to the left of the image.

(c) Location: SZ2

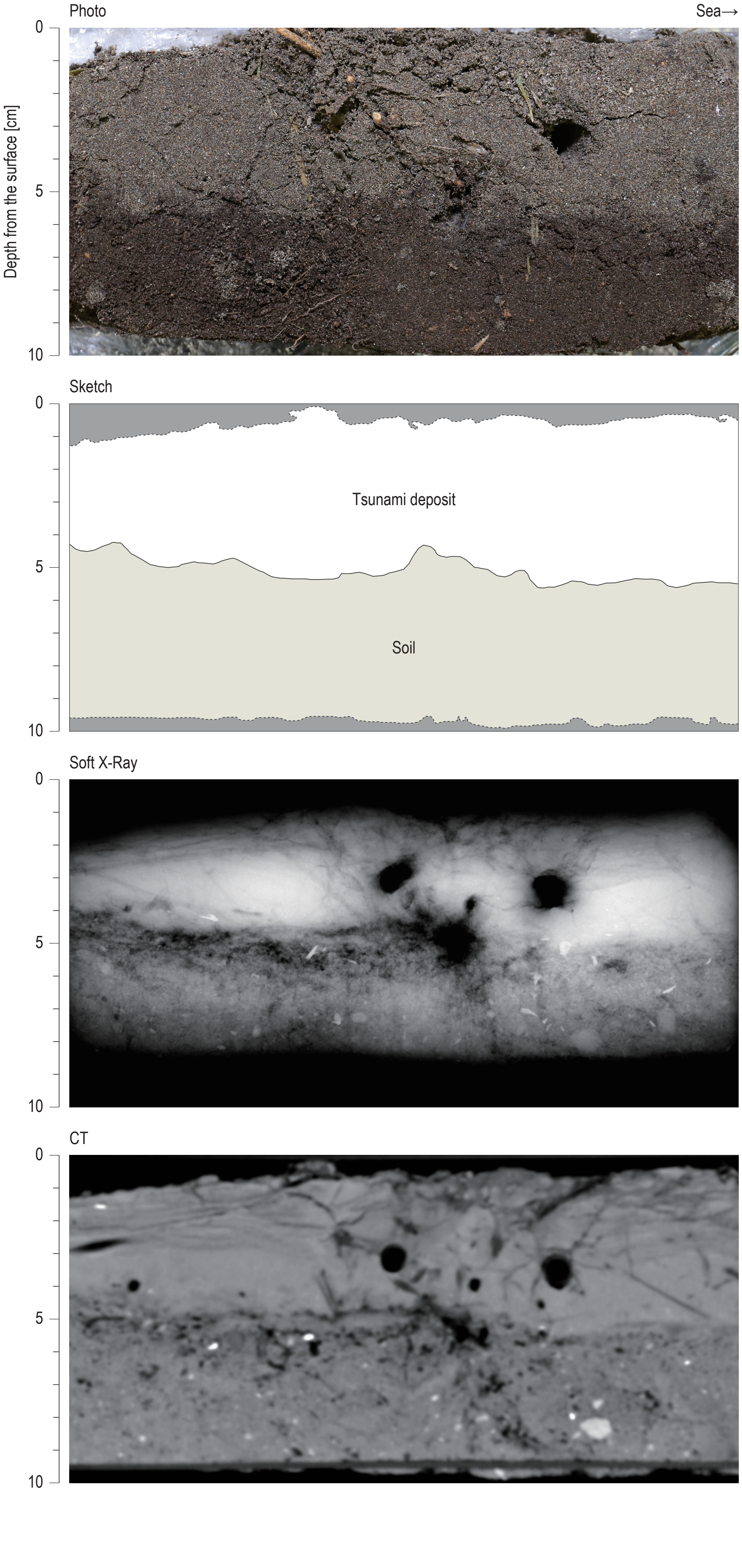

Shimada et al. Supplementary Data S3 *Continued*
Photographs, sketches, soft X-ray images, and CT images of the tsunami deposits. (a) at SZ1, (b) trench photograph at SZ1, (c) at SZ2, (d) at SZ3, (e) at SZ4, (f) at SZ5. Note that the sea direction is to the right in all images except the photograph of the trench, in which the sea is to the left of the image.

(d) Location: SZ3

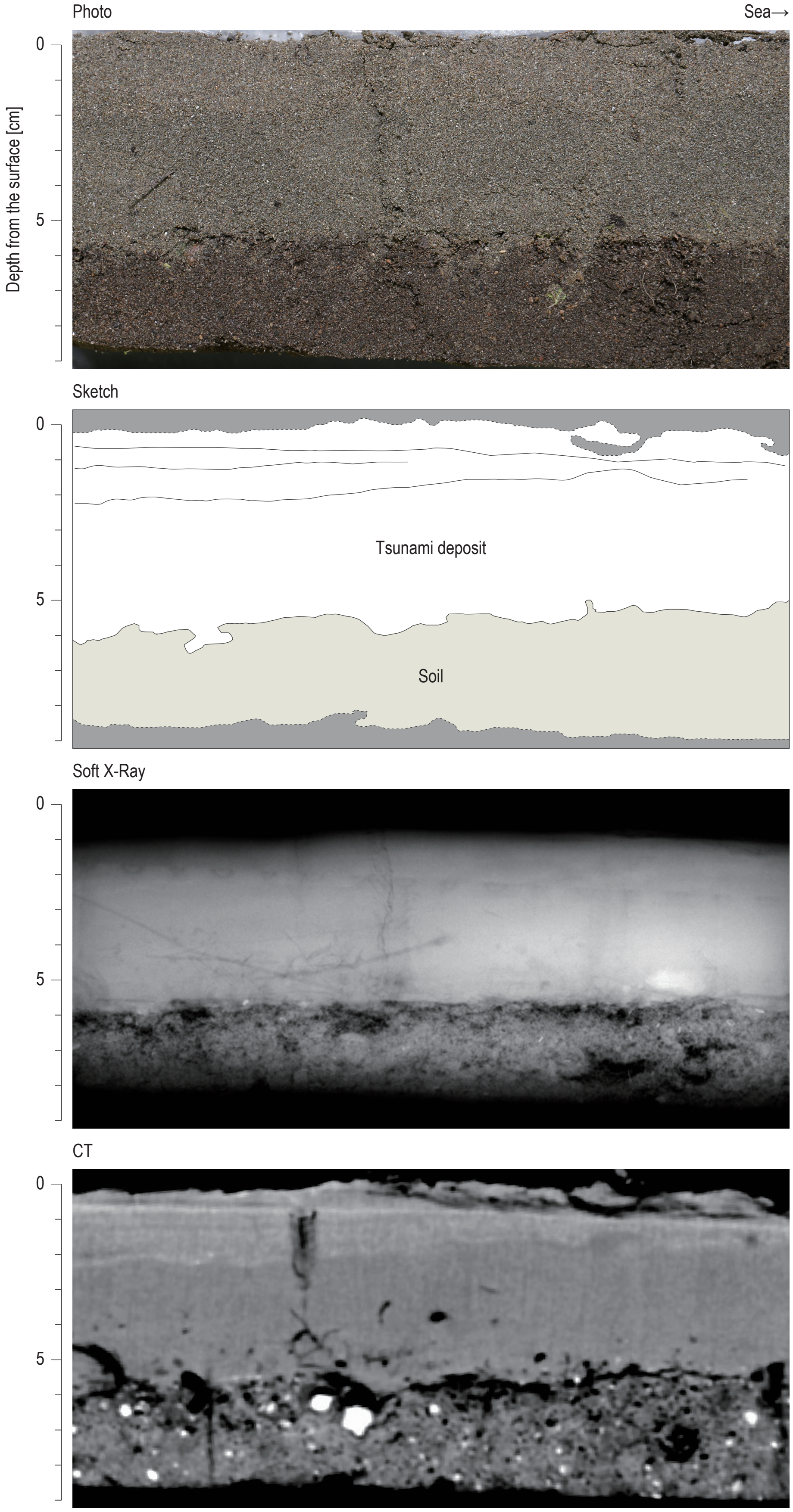

Shimada et al. Supplementary Data S3 *Continued*
Photographs, sketches, soft X-ray images, and CT images of the tsunami deposits. (a) at SZ1, (b) trench photograph at SZ1, (c) at SZ2, (d) at SZ3, (e) at SZ4, (f) at SZ5. Note that the sea direction is to the right in all images except the photograph of the trench, in which the sea is to the left of the image.

(e) Location: SZ4

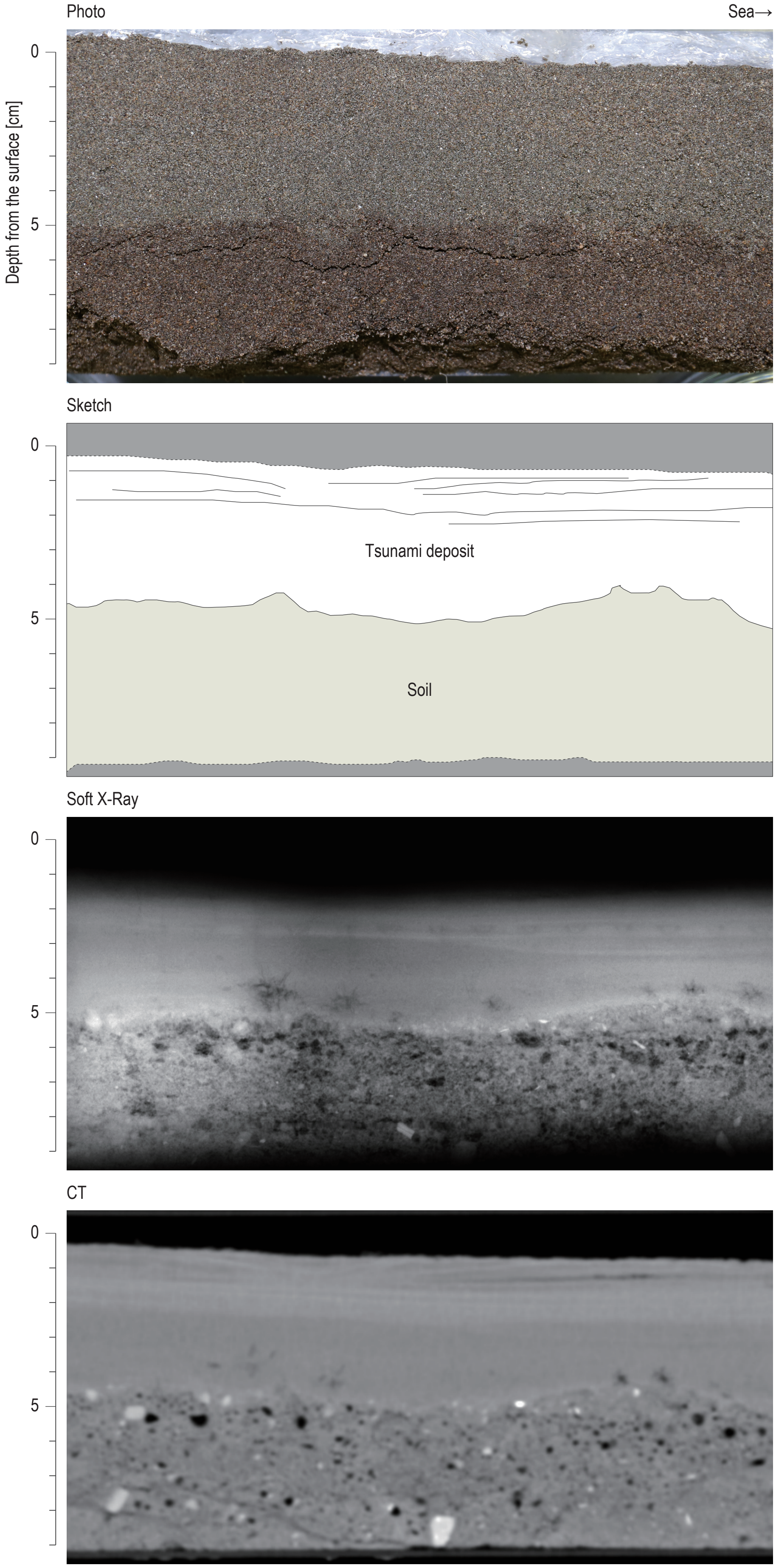

Shimada et al. Supplementary Data S3 *Continued*
Photographs, sketches, soft X-ray images, and CT images of the tsunami deposits. (a) at SZ1, (b) trench photograph at SZ1, (c) at SZ2, (d) at SZ3, (e) at SZ4, (f) at SZ5. Note that the sea direction is to the right in all images except the photograph of the trench, in which the sea is to the left of the image.

(f) Location: SZ5

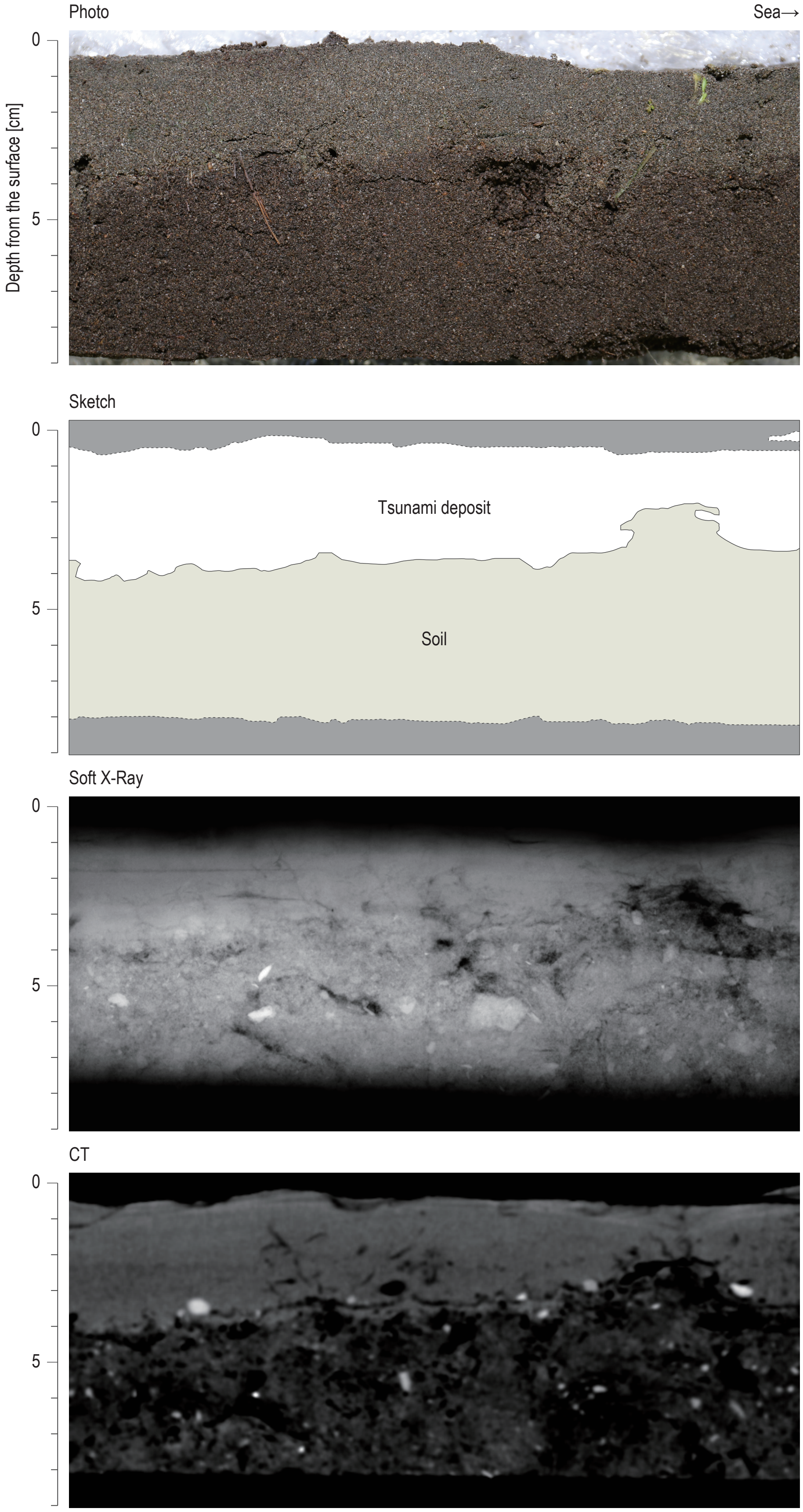

Supplement: Supplementary file 4 — Supplementary Data S3. [file 41598_2025_90945_MOESM4_ESM.pdf]
